# Glutamine Deprivation Induces Abortive S-Phase Rescued by Deoxyribonucleotides in *K-Ras* Transformed Fibroblasts

**DOI:** 10.1371/journal.pone.0004715

**Published:** 2009-03-05

**Authors:** Daniela Gaglio, Chiara Soldati, Marco Vanoni, Lilia Alberghina, Ferdinando Chiaradonna

**Affiliations:** Department of Biotechnology and Biosciences, University of Milano-Bicocca, Milan, Italy; Ordway Research Institute, United States of America

## Abstract

**Background:**

Oncogene activation plays a role in metabolic reprogramming of cancer cells. We have previously shown that *K-ras* transformed fibroblasts have a stronger dependence on glycolysis and a reduced oxidative phosphorylation ability as compared to their normal counterparts. Another metabolic adaptation of cancer cells, that has long been established, is their propensity to exhibit increased glutamine consumption, although the effects induced by glutamine deprivation on cancer cells are still controversial.

**Methodology and Principal Findings:**

Here, by using nutritional perturbations and molecular physiology, we show that reduction or complete depletion of glutamine availability in *K-ras* transformed fibroblasts causes a strong decrease of proliferation ability and a slower re-entry of synchronized cells into the cell cycle. The reduced proliferation is accompanied by sustained expression of cyclin D and E, abortive S phase entrance and is dependent on Ras signalling deregulation, since it is rescued by expression of a dominant negative guanine nucleotide exchange factor. The growth potential of transformed cells as well as the ability to execute the G_1_ to S transition is restored by adding the four deoxyribonucleotides, indicating that the arrest of proliferation of *K-ras* transformed cells induced by glutamine depletion is largely due to a reduced supply of DNA in the presence of signalling pathways promoting G_1_ to S transition.

**Conclusions and Significance:**

Our results suggest that the differential effects of glutamine and glucose on cell viability are not a property of the transformed phenotype *per se*, but rather depend on the specific pathway being activated in transformation. For instance, *myc*-overexpressing cells have been reported to die under glutamine depletion and not under glucose shortage, while the opposite holds for *ras*-transformed fibroblasts as shown in this paper. These different responses of transformed cells to nutritional stress should be taken into account when designing anti-cancer therapies that aim to exploit metabolic differences between normal and transformed cells.

## Introduction

Increasing attention has been given in recent years to the connection between metabolic alterations and cancer [1]. Under aerobic conditions, normal cells use oxidative phosphorylation as a predominant source for ATP generation. In sharp contrast to normal cells, a common feature of most cancer cells is a major use of glycolysis to produce ATP [2]–[6]. The onset of a glycolytic phenotype and/or impaired mitochondrial function [7]–[9] in cancer cells have been reported to derive from several mechanisms, including adaptation to hypoxia, oncogene activation or loss of anti-oncogene and the consequences of glucose deprivation have been extensively described in several cancer cells and tissues, for a review see [10]. Another metabolic adaptation of cancer cells, that has long been established, is their propensity to exhibit increased glutamine consumption, although the effects induced by glutamine deprivation on cancer cells are still controversial, mostly because of the diverse cellular processes in which glutamine is involved. Indeed, as a metabolic precursor glutamine is required for protein, RNA and DNA biosynthesis, and through glutaminolysis, participates in energy production and cellular redox homeostasis, especially to glutathione synthesis [11]. Accordingly, different Authors assigned to glutamine a role in: growth and survival of several cell lines such as fibroblasts, enterocytes, and lymphocytes through modulation of the signal transduction pathway(s) and the cell cycle machinery; suppression of apoptosis through a modulation of the ability to respond to stress; maintenance of metabolic processes such as TCA cycle, fatty acid synthesis controlling the supply of essential intermediates. Therefore, understanding the effects of glutamine deprivation is necessary to obtain a more complete appreciation of changes in tumor metabolism [11].

NIH3T3 cells are a genetically well defined immortalized cell line that has long established as a model parental cell line for the study of cell transformation [12], [13]. Like some other cancer cells, *K-ras* transformed fibroblasts exhibit a high rate of glucose consumption associated with mitochondrial dysfunction and deregulated transcription of several mitochondrial genes [14], [15] and unpublished results, events often associated with cancer phenotype. As a result, *K-ras* transformed NIH3T3 cells are remarkably sensitive to glucose deprivation [15], a condition in which they stop growth and die. Transformation-related phenotypes of *K-ras* transformed cell lines can be rescued by expression of a dominant-negative guanine nucleotide exchange factor (GEF-DN) [15]–[17].

Here we compared the physiological response to glutamine limitation of normal NIH3T3 mouse fibroblasts (normal cells); NIH3T3 cells transformed by an activated form of the *K-ras* oncogene (transformed cells) and *K-ras* transformed NIH3T3 fibroblasts reverted by expression of a GEF-DN (reverted cells). Glutamine deprivation strongly decreases proliferation of transformed cells, while having little, if any, effect on normal and reverted lines. No glutamine depletion-dependent reduction in overall protein synthesis or in ATP level was observed in transformed cells compared to their isogenic counterpart. Reduced proliferation of transformed cells was accompanied by sustained accumulation of cyclin D, E and A and abortive S phase entrance. The proliferation defect of transformed cells could be restored by adding the four deoxyribonucleotides (but not TCA cycle intermediates), indicating that the arrest of growth of *K-ras* transformed cells induced by glutamine depletion is largely due to a reduced supply of DNA precursors in the presence of active signaling pathways promoting entrance into S phase.

## Results

### Reduced proliferation of K-ras transformed fibroblasts in media containing low initial glutamine concentration is associated to an increased fraction of cells in S-phase

Glutamine is an important substrate for several cellular processes. We tested whether lowering initial glutamine concentration in culture medium elicited differential effects on the proliferation of transformed cells as compared to normal cells. Asynchronous normal and transformed cell lines were cultured in normal growth medium (4 mM glutamine), in an intermediate medium (1 mM glutamine) and in a low glutamine medium (0.5 mM glutamine). These concentrations were chosen considering glutamine levels normally used in cell culture (between 4 and 2 mM) as well as that determined in human plasma (0.6 mM). All media were supplemented with 25 mM glucose. Cells were followed for at least 144 hours, that is, from the moment of seeding to when they either reached confluence, started to grow in multi-strata or to die. All experiments reported in this and the following paragraphs refer to the above-mentioned experimental setup.

**Figure 1:**
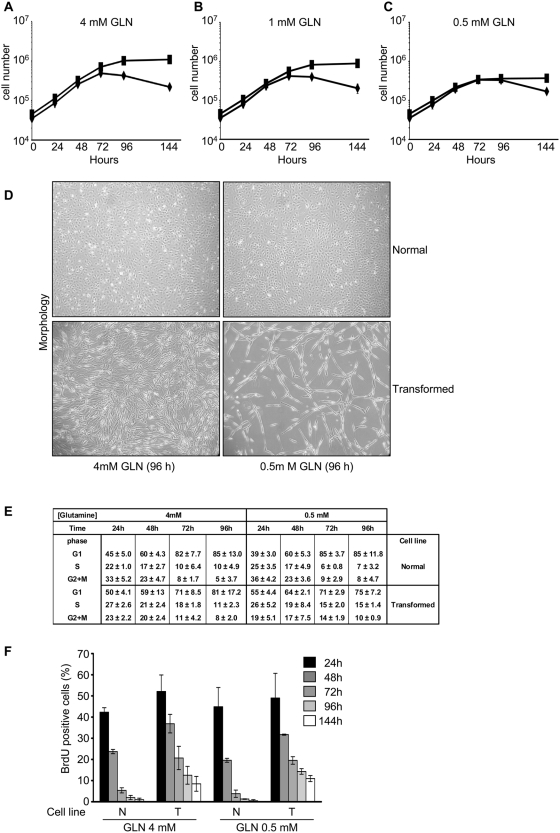
Glutamine shortage induces cell proliferation arrest and S-phase accumulation in oncogenic *K-ras* transformed fibroblasts. Normal (⧫) and transformed (▪) cell lines were plated at 3000 cells/cm^2^ in 6-well plates in normal medium. Culture medium was replaced after 24 hours with a normal medium −4 mM glutamine- (A), an intermediate medium −1 mM glutamine- (B) and a low medium −0.5 mM glutamine- (C) and the cells were collected and counted at indicate time. (D) Morphological analysis of normal cells (upper images) and transformed cells (lower images) cultured in 4 mM glutamine (left images) and 0.5 mM glutamine (right images) at 96 hours. Cells were observed at phase-contrast microscopy. Exponentially growing normal and transformed cells were incubated for increasing periods of time (24, 48, 72, 96 and 144 h) in medium containing 4 mM and 0.5 mM glutamine, then harvested, counted and subjected to FACS analysis after staining with propidium iodide (E), or pulsed 30 minutes with BrdU and then analyzed by fluorescence microscopy using an anti-BrdU specific antibody (F). Results are the mean of at least triplicate determinations with standard deviations indicated. For BrdU staining were analyzed at least 200 cells for each sample.

Normal cells stopped growth after 72 hours, regardless of glutamine concentration. At later time, cell number started to decrease (Fig. 1A–C, ⧫ symbol). Concurrently, apoptotic phenotypes - including the presence of floating, dead cells (Figure 1D, upper panels) - were observed in normal cells regardless of glutamine concentration, possibly because of prolonged contact inhibition. In normal and intermediate glutamine medium, transformed cells continued to proliferate and reached a much higher cell density than normal cells, but proliferation advantage of transformed cells was almost completely lost when they were grown in low glutamine medium as scored by both cell counting (Fig. 1C, ca 1,1×10^6^, 0,9×10^6^ and 0,4×10^6^ at 144 hours respectively for cells grown in normal, intermediate and low glutamine concentration) and direct microscopic observation (Fig. 1D, compare lower left and lower right bottom panels). Regardless of glutamine concentration, little, if any, floating, dead cells were observed in transformed cells (Fig. 1D). A similar loss of proliferation potential was observed in the human HEP3B and HCT116 cancer cell lines when cultured in 0.5 mM glutamine (data not shown), suggesting that the inhibition of cell proliferation by glutamine reduction is a general feature of transformed cells.

As previously reported [15], balanced exponential phase was very short, since despite exponential increase in cell number, the fraction of G_1_ cells started to increase as early as at 48 hours after the beginning of the experiment, the more so for normal cells (Fig. 1E). Remarkably, transformed cells grown in low glutamine medium retained quite a large fraction of S and G_2_/M phase cells even at the 72 or 96 hours time points, *i.e.*, when the increase in cell number had stopped. Indeed, after 72 hours of growth, transformed cells had a very similar fraction of cells in the S+G_2_M phases regardless of glutamine concentration, while a significant difference could be observed compared to normal cells. Quantification of the amount of BrdU incorporated into DNA is taken to be more precise than Propidium Iodide to evaluate S-phase cells, therefore DNA synthesis was then analyzed by BrdU incorporation (30 min pulse) detected by immunofluorescence using a BrdU specific antibody and BrdU-positive cells were counted (Figure 1F). While the fraction of normal BrdU-incorporating cells steadily decreased from 48 hours, arriving to around 1% after 96–144 hours of growth, the fraction of transformed BrdU-incorporating cells at 96–144 hours of growth remained high, the more so in cells growing in media supplemented with 0.5 mM glutamine. Compare for instance normal and transformed cells grown for 96 hours in media supplemented with 0.5 mM glutamine: normal and transformed cells have reached the same, maximal density, but the fraction of BrdU-incorporating cells is almost 0% for normal cells and at least 15% in transformed cells. This value remains constant until the end of the experiment and is essentially the same for cells grown in 4 mM or 0.5 mM glutamine, despite the large difference in proliferation.

Glutamine uptake was indirectly measured by assaying residual glutamine in the medium (Figure S1A and B). At each glutamine availability, normal and transformed cells consumed glutamine at the same rate. Remarkably, glutamine uptake was much faster in cells grown at low initial glutamine concentration (Figure S1B). We could show that at least 20% of initial glutamine (i.e. ca 0.8 mM) is still present at 96 hours in cells grown at 4 mM initial glutamine concentration, i.e. when normal cells have stopped to proliferate (Figure S1A). In normal and transformed cells grown in low initial glutamine concentration, on the contrary, no residual glutamine is present at 96 hours. Transformed cells stopped growth afterwards, indicating that glutamine acts as a limiting nutrient under these conditions. We could neither detect any glutamine in Newborn Calf Serum nor see any difference in growth when growth media were supplemented with dialyzed serum (data not shown).

When initial plating density was lowered to 2000 cells/cm^2^ (compared to our standard plating density of 3000 cells/cm^2^) the same phenotypes reported above were observed, but with a 24 hour delay, consistently with the notion that glutamine is not the factor limiting proliferation of normal cells in our experimental set-up (Figure S2A, C and D).

### In transformed cells sustained S-phase in low glutamine is associated with prolonged expression of cyclin D, E and A, enhanced pRb phosphorylation, decreased level and cytoplasmic localization of p27^Kip1^

Oncogenic Ras activation is known to promote the G_1_ to S progression [15]–[18], [19]. Results reported in the previous paragraph indicate that transformed cells grown in low glutamine medium retain a large fraction of cells in S-phase while proliferation is stopped suggesting that in transformed cells glutamine depletion does not efficiently shut-down the G_1_ to S. It was therefore of interest to analyze both in normal and in transformed cells modulation by glutamine of relevant parameters, such as level, phosphorylation state and sub-cellular localization of proteins involved in the G_1_ to S transition.

Transcription of genes required for the onset of S-phase in mammalian cells is induced by the E2F/DP transcription factors, whose activity in early G_1_ cells is down-regulated by the pRb protein. Release of inhibition by pRb requires its phosphorylation by upstream cyclin dependent kinase complexes, namely Cdk4/cyclin D and Cdk2/cyclin E [20]. Notably both Cdk2 and Cdk4 kinase complexes phosphorylate, during the G_1_ to S transition, pRb protein on Ser 795. Several Authors have considered this phosphorylation as a readout of Cdks activity. D-type and E-type cyclin/Cdk complexes are regulated by cyclin binding, by phosphorylation and by two families of Cdk inhibitors: the INK4 family, that acts specifically on Cdk4, and the KIP family, that comprises p21^Cip1^, p27^Kip1^ and p57^Kip2^ acting both on Cdk4 and Cdk2 [21]. Besides, p21^Cip1^ and p27^Kip1^ also facilitate assembly and activation of cyclin D/Cdk4 in early G_1_ [21], [22].

**Figure 2:**
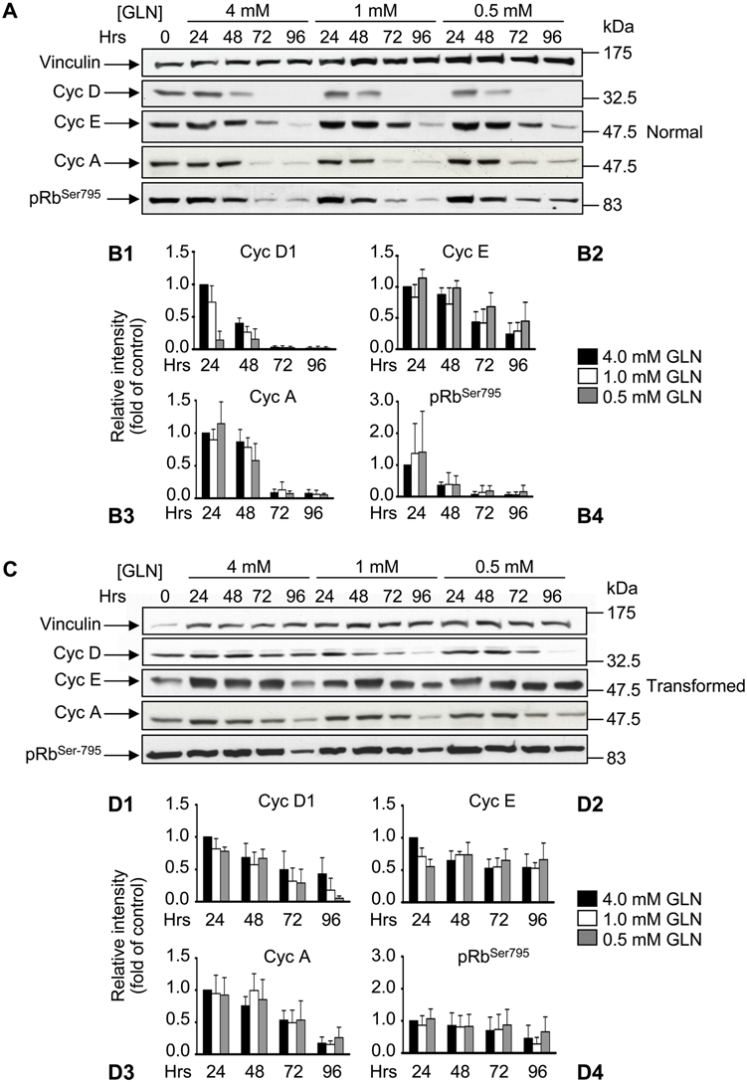
Expression of proteins involved in the G_1_ to S transition in normal and transformed cells. For the protein expression analysis normal cells (A) and transformed cells (C) grown in media containing 4 mM glutamine, 1 mM glutamine and 0.5 mM glutamine were collected at appropriate time points and 40 µg of proteins from the total cellular extract were subjected to SDS-PAGE followed by Western blotting with appropriate antibodies. One of at least three independent experiments is shown. (B1–B4 and D1–D4) Relative quantitative analysis of protein levels after Western blot film acquisition by scanner and densitometry analysis using Image J program. The densitometry values obtained for each protein were normalized by using the values of the corresponding vinculin and were plotted considering the densitometry value obtained for the sample 24 hours/4 mM glutamine as equal 1. Results are the mean of at least triplicate determinations with SD indicated.

**Figure 3:**
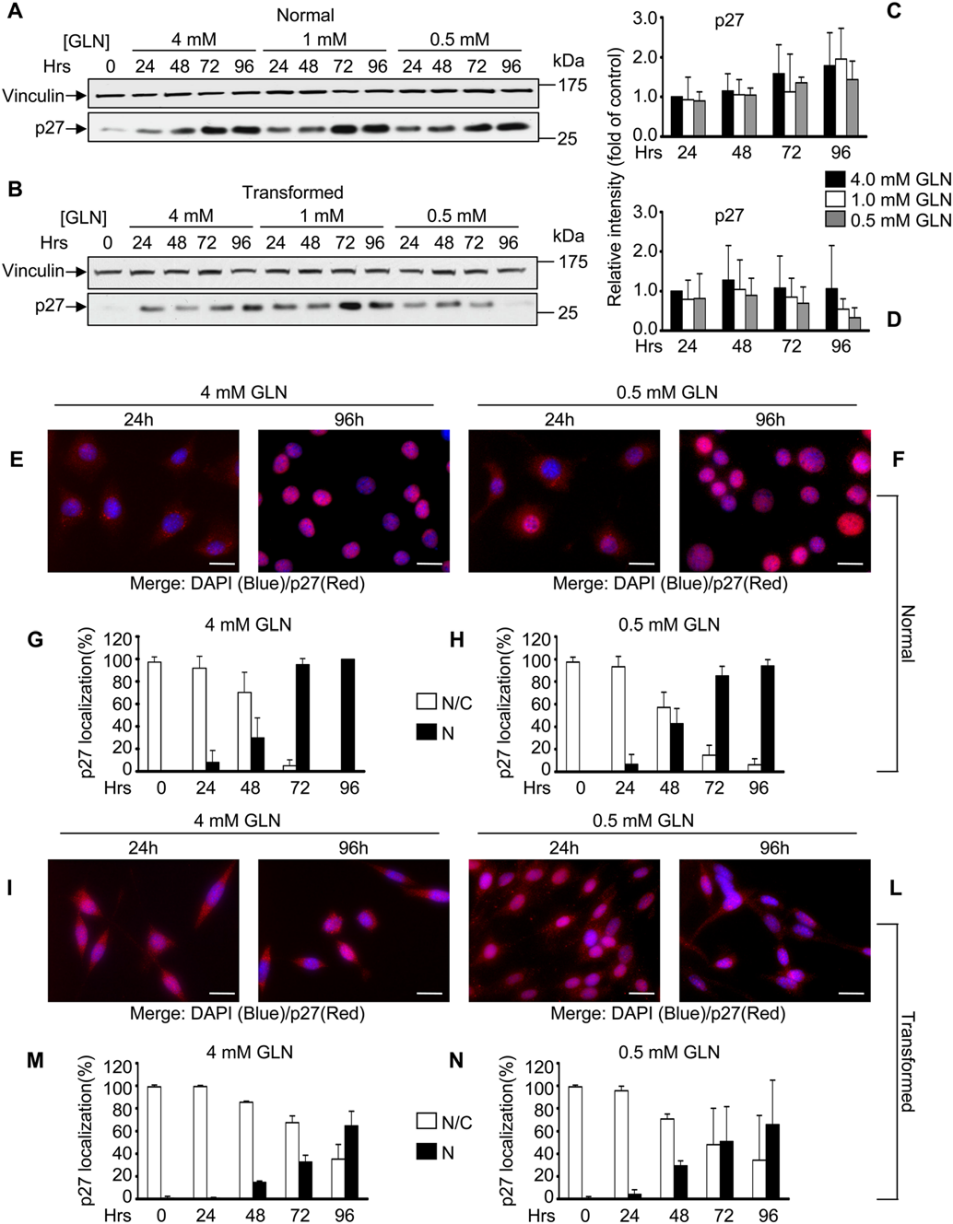
Expression and localization of p27^kip1^ in normal and transformed cells. For p27^kip1^ expression analysis normal cells (A) and transformed cells (B) grown in media containing 4 mM glutamine, 1 mM glutamine and 0.5 mM glutamine were collected at appropriate time points and 40 µg of proteins from the total cellular extract were subjected to SDS-PAGE followed by Western blotting with p27^kip1^ specific antibody. One of at least three independent experiments is shown. Relative quantitative analysis of p27^kip1^ protein levels after Western blot film acquisition by scanner and densitometry analysis using Image J program, as described in Figure 2, in normal cells (C) and transformed cells (D). p27^kip1^ cellular localization in normal (E, F, G and H), and transformed (I, L M and N) cell lines was analyzed by immunofluorescence staining for p27^kip1^ (red staining), after 24 and 96 hours of growth in media supplemented with either 4 mM glutamine (left pictures) or 0.5 mM glutamine (right pictures). Nuclei were visualized by DAPI staining (Blue staining). The pictures are the result of a merge between the two single images acquired. At least 200 cells were scored for each sample. The averages of three independent experiments are presented in G, H, M and N with errors bars indicating SD. Bars, 20 µm.

pRb phosphorylated on Ser795 was detected using a phospho-specific antibody raised against pRb^Ser795^ and that does not cross react with unphosphorylated pRb. In normal cells, and regardless of initial glutamine concentration, Ser795-phosphorylated pRb - *i.e.* the form that monitors the activity of both Cdk4 and Cdk2 kinases - decreases as cells approach and reach confluence (Figure 2, panels A and B4). Concurrently, drops in cyclin D1, E and A were observed, their level being at - or below - the detection limit at the 96 hours time point (Figure 2, panels A through B3). A decrease in Cdk2 and a more permanent expression of Cdk4 was observed during the time course of the experiment (Figure S3). Moreover, as shown in Figure 3A, 3C and S4B and S4C, in normal cells grown in media supplemented with the three initial glutamine concentrations, the expression of the p27^Kip1^ and p21^Cip1^ proteins showed a time-dependent increase for p27^Kip1^ and a decrease for p21^Cip1^ (independent of the initial glutamine concentration).

The inhibitory function of p27^Kip1^ is regulated not only through synthesis and degradation, but also by sub-cellular localization. Indeed, cytoplasmic p27^Kip1^ has been detected in about 40% of primary human breast cancers in conjunction with Akt activation and is associated with poor patient prognosis and reduced Cdks inhibitory activity [23]–[25]. In normal cells, grown in 4 and 0.5 mM GLN, associated with the time-dependent increase of p27^Kip1^, we observed that p27^Kip1^ staining remained diffuse at early time points (24–48 hours - proliferating cells -, Figure 3, panels E, F, G and H), and strongly accumulated in the nucleus at later time points (72–96 hours- arrested cells – Figure 3, panels E, F, G and H). Increased p27^Kip1^ expression and nuclear localization well correlate with the decrease of S-phase cells and of pRb^Ser795^ phosphorylation observed during the time course of the experiment.

For transformed cells the pattern was very different. In cells grown in media supplemented with 4 mM glutamine, Cyclin D, A and E as well as phospho-Rb decreased only to about 50% of the level present at 24 hours (Figure 2, panels C through D4), while Cyclin D1 decreased significantly more at intermediate and low glutamine concentrations. Differently to the pattern observed in normal cells, transformed cells showed fairly constant Cdk4, Cdk2 and p21^Cip1^ levels during the time course of the experiment (Figure S3 and S4C), and no increase in the level of p27^Kip1^ (Figure 3D): indeed p27^Kip1^ was barely detectable in cells grown in low glutamine, at the last time point (96 h) (Figure 3B and D). Immunofluorescence staining, in both low and high glutamine availability, revealed that in transformed cells a large fraction of p27^Kip1^ remained cytoplasmic, in contrast to the situation observed in normal cells where the majority of this Cki is nuclear at later time points (compare Figure 3 I–N, for transformed cells with Figure 3E–H, for normal cells). However, double immunofluorescence staining performed by using anti-BrdU and anti-p27^Kip1^ specific antibodies, to score p27^Kip1^ localization in normal and transformed BrdU positive cells, showed that almost all the BrdU positive cells had a nucleo/cytoplasmic localization of p27^Kip1^ protein (Figure S4A and B).

### Down-regulation of Ras signalling by a dominant-negative Ras-specific GEF reverts low glutamine-dependent phenotype in K-ras transformed fibroblasts

**Figure 4:**
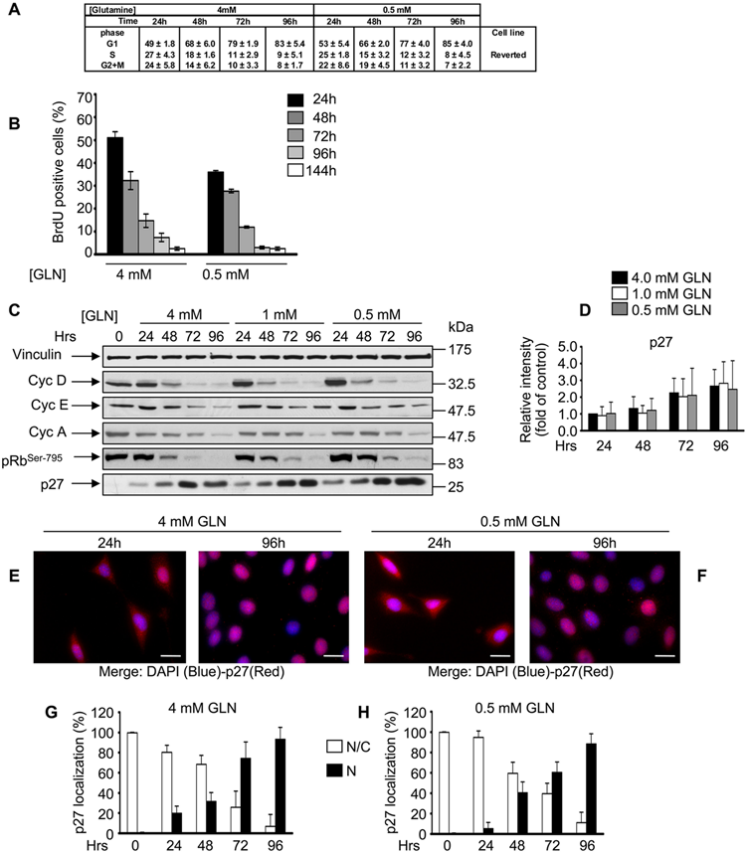
The GEF-DN protein completely reverts the phenotype induced by oncogenic *K-ras* expression in glutamine shortage. Cell cycle distribution (A), S-phase cell population (B), time-course of the expression of proteins involved in the G_1_ to S transition and pRb phosphorylation in Serine795 (C), relative quantitative analysis for p27^kip1^ protein (D) and p27^kip1^ cellular localization (E, F, G and H) in reverted cells grown in diverse initial glutamine availability as differently indicate in the Figure. The experiments were performed as indicated in previous figures. The averages of at least three independent experiments are presented in A, B, D, G and H with errors bars indicating SD. Bars, 20 µm.

Overexpression of a dominant negative Ras-specific GEF protein has been reported to phenotypically revert several phenotypes in *K-ras* transformed murine fibroblasts, including Ras-GTP level, morphology, anchorage independent growth, reduction of Ras-dependent tumor formation in nude mice, glucose dependence and mitochondrial dysfunction [15]–[17]. As shown in Figure 4, *K-ras* transformed cells expressing the dominant-negative GEF showed a complete reversion of all the phenotypes observed in transformed cells grown in all the three glutamine conditions. Indeed, as shown in Figure 4A and B, reverted cells accumulated in G_1_, decreased the fraction of BrdU positive cells, reduced their proliferation ability (data not shown) in time-dependent manner, as observed in normal cells. Such results were totally confirmed by protein expression analysis (Figure 4D) in which a decrease of cyclin D1, E, A and pRb phosphorylation and an increase of p27^Kip1^ in a time-dependent and glutamine-independent manner were observed. Finally, cellular localization analysis of p27^Kip1^ protein showed a similar behaviour to that observed in normal cells (Figure 4E, F, G and H). Taken together, these data support the notion that the exquisite sensitivity of transformed cells to glutamine depletion is due to constitutive expression of oncogenic K-ras.

### Glutamine reduction does not substantially interfere with RNA synthesis, protein synthesis and energy metabolism

Amino acid deprivation leads to a fall in the rate of RNA and protein synthesis and consequently to a reduction of total cellular protein content [26], [27]. Such effect could be even more significant upon depletion of glutamine - the more abundant amino acid in tissues, body fluids and cell culture medium - because of its involvement in the biosynthesis of several amino acids (glutamate, aspartate, arginine, alanine) as well as in energy production [28]–[30] and nucleotide biosynthesis [31].

**Figure 5:**
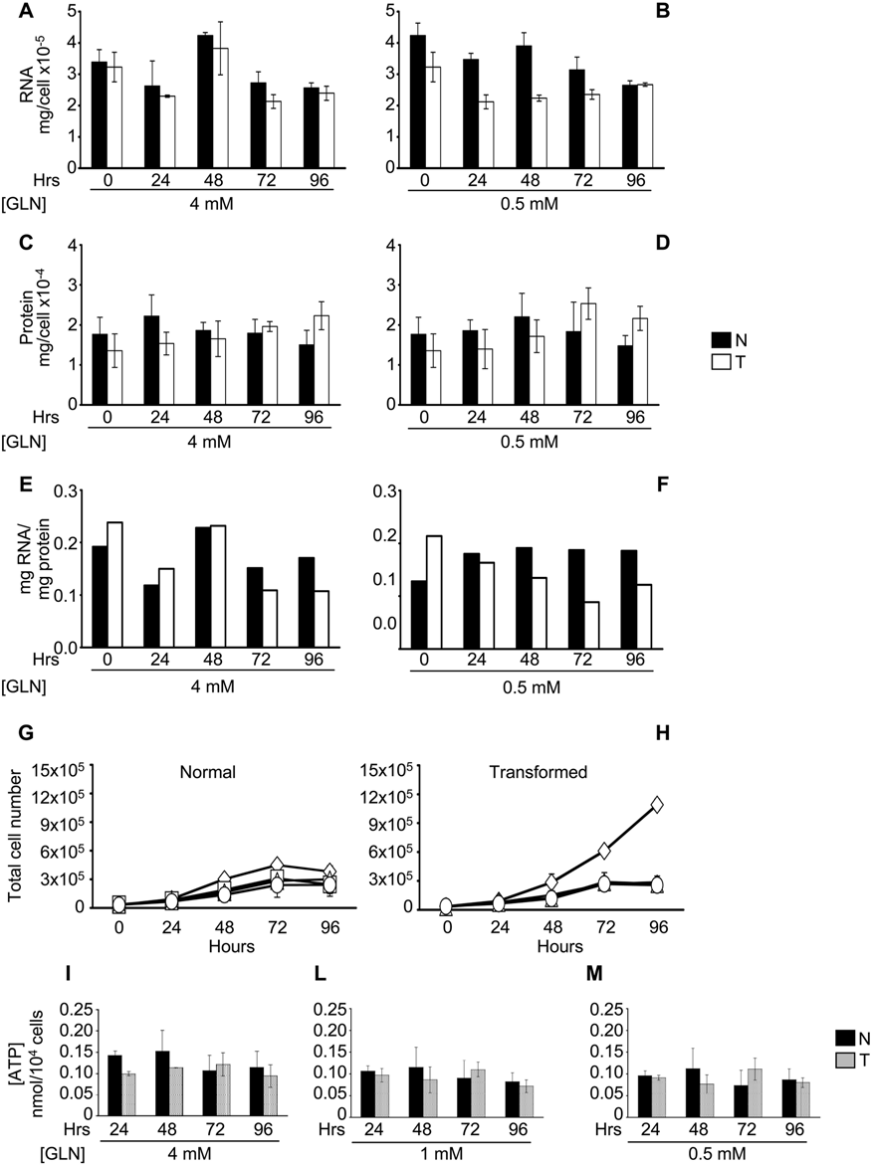
Analysis of some glutamine-dependent cellular processes in normal and transformed cells. Amount of the total RNA, proteins and ratio RNA/protein for single cell calculated after spectrophotometric determination of total RNA extracted by using TRIzol solution and different lysis buffer (protein) of normal and transformed cells grown in 4 mM glutamine (A, C and E), and 0.5 mM glutamine (B, D and F) along a time course. Normal (G) and transformed (H) cell lines were plated at 3000 cells/cm^2^ in 6-well plates in normal medium. Culture medium was replaced after 24 hours with a normal medium −4 mM glutamine- (◊), a low medium −0.5 mM glutamine- (□), a medium with 0.5 mM glutamine and 2 mM pyruvate (▵) and a medium with 0.5 mM glutamine and 2 mM malate (○) and then the cells were collected and counted at indicate time. ATP content in the two cell lines was measured using a luciferin-luciferase assay along the time course in 4 mM (I), 1 mM (L) and 0.5 mM glutamine availability (M). ATP concentrations were calibrated against ATP standards and expressed as nanomoles per 10.000 cells. In all the experiments the averages of at least three independent experiments are presented with errors bars indicating SD.

We first analyzed whether glutamine deprivation could affect total RNA and protein accumulation. To this end, the time course of average of RNA and protein content per cell was analyzed. The average RNA content of normal and transformed cells, regardless of glutamine availability, slightly decreased along the time course of the experiment. The time course decrease in RNA content of transformed cells growing in low glutamine, was somehow more sizeable as compared to that observed in normal cells suggesting that glutamine reduction may hamper biosynthesis of nucleotides and hence the synthesis of stable RNA (Figure 5A and B).

As cells approached and reached stationary phase, and regardless of glutamine concentration, a small decrease in protein content was observed in normal cells (Figure 5C and D). On the contrary, the protein content of transformed cells showed a substantial increase with time. No major quali-quantitative changes could be observed by Comassie staining of SDS-PAGE gels, at least within the limited resolution of this technique (Figure S5). These results are summarized in panels E and F, showing that in 4 mM glutamine, time-dependent changes in the RNA/protein ratio for normal and transformed cells are very similar, while transformed cells show a more detectable decrease of this parameter when grown under limiting glutamine.

Glutamine is an oxidizable fuel, which enters the TCA cycle as α-ketoglutarate, by reactions catalyzed by aspartate amino transferase (AST) and by glutamate dehydrogenase [32], [33]. Accordingly it has been reported that glutamine depletion could cause a deficiency of this cycle [34], [35]. Supplementing the growth medium (containing 0.5 mM glutamine) with either 2 mM pyruvate, which can enter the TCA cycle mainly through its conversion in Acetyl-CoA or 2 mM malate which can enter the TCA cycle by conversion into oxaloacetate and/or furnish reducing equivalents by malic enzyme activity, in the last steps of glutaminolysis, did not rescue the proliferation defect caused by low glutamine in transformed cells (Figure 5G and H). Indeed the three growth curves, corresponding to 0.5 mM glutamine alone or plus pyruvate or malate were completely superimposed for both cell lines.

The ATP level was measured in normal and transformed cells (24, 48, 72 and 96 hours in 4, 1 and 0.5 mM glutamine, Figure 5I, L and M). ATP decreased in both cell lines along the time-course of the experiment. In all cases substantial residual ATP was present even at the latest assayed time, however normal cells showed a more pronounced drop in ATP (20%, 41% and 38% for cells grown in media supplemented with 4 mM, 1 mM and 0.5 mM initial glutamine, respectively) than transformed cells (5%, 25% and 18% for cells grown in media supplemented with 4 mM, 1 mM and 0.5 mM initial glutamine, respectively). The same analyses performed on reverted cells confirmed a behavior similar to that observed in normal cells (data not shown). These results rule out the possibility that glutamine effects on transformed cells are due to energy depletion.

### Addition of deoxyribonucleotide triphosphates rescues the abortive S-phase entrance induced by low or full absence of glutamine in K-ras transformed fibroblasts

**Figure 6:**
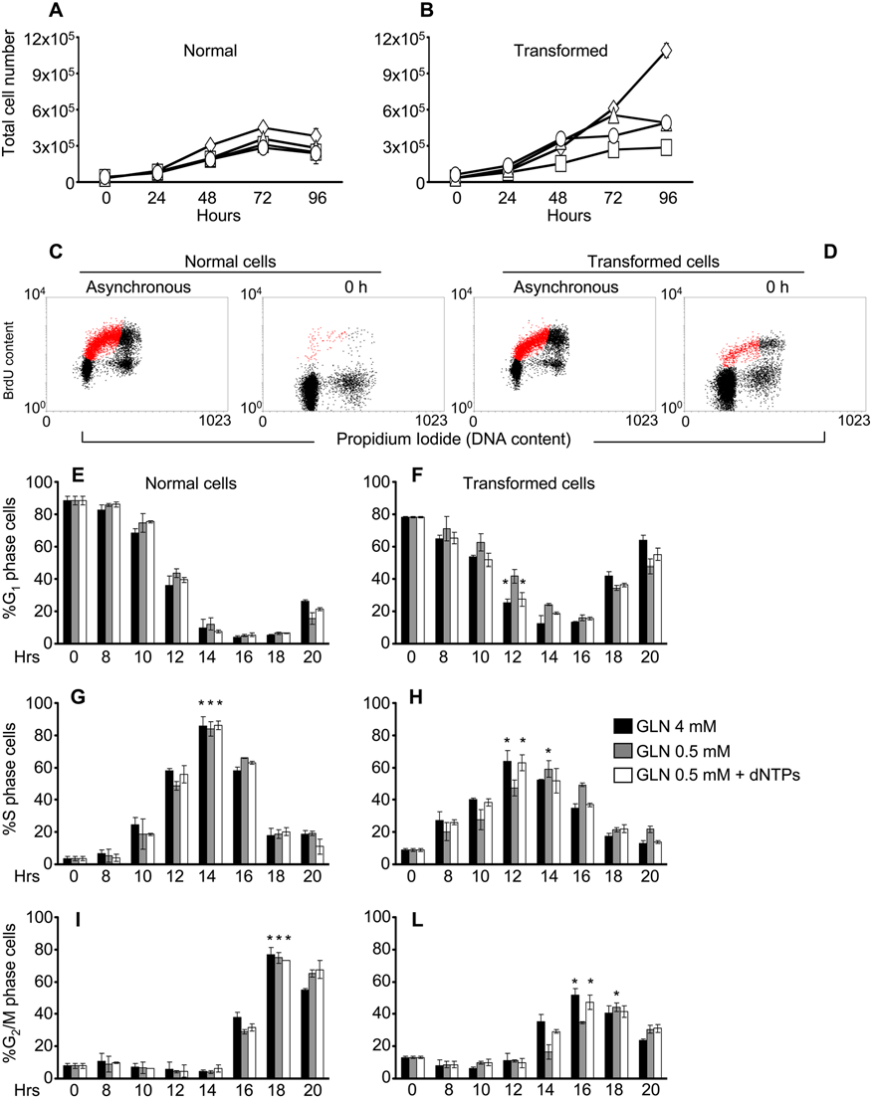
Proliferation analysis of normal and transformed cells adding in culture medium the four dNTPs. Normal (A) and transformed (B) cell lines were plated as previously described. Culture medium was replaced after 24 hours (indicated as 0 h) with a normal medium −4 mM glutamine- (◊), a low medium −0.5 mM glutamine- (□), a medium with 0.5 mM glutamine and 10 µM dNTPs -ATP, GTP, TTP and CTP- (▵) and a medium with 0.5 mM glutamine and 10 µM dNTPs in which the dNTPs were added at 0 and 48 hours (○), then the cells were collected and counted at indicate time. Normal (left panels) and transformed (right panels) cells, synchronized by 24 hours serum starvation, were released in a medium containing 10% serum, 4 mM or 0.5 mM glutamine, dNTPs and BrdU for continuous DNA labeling. At indicate time points the cells were collected and stained with propidium iodide and anti-BrdU specific antibody and analyzed by flow cytometry. Representative cytograms, obtained for asynchronous and 0 hours (time 0) time point samples respectively, plotting BrdU content (labeled cells) *vs.* PI staining (DNA content) (Figure 6 C - normal cells - and D - transformed cells -). Red color in the plot represents the S-phase cells. The percentages of cells in G_1_ (panels E and F), S (panels G and H) and G_2_/M (panels I and L), phase of normal (left panels) and transformed (right panels) cells released in 4 mM and 0.5 mM glutamine alone or plus dNTPs obtained by bi-parametric analysis as shown above, are represented. The asterisks indicate the S or G_2_/M phase peaks identified in both glutamine availability. The error bars correspond to standard deviations of triplicate analysis.

Results reported above indicate that in transformed cells energy metabolism under limiting glutamine concentration is not heavily unbalanced, since these cells have normal ATP and protein levels, and do not benefit from increasing fuel supply to the TCA cycle. On the other hand under limiting glutamine conditions, transformed fibroblasts fail to down-regulate the machinery responsible for the G_1_ to S transition, as evidenced by sustained accumulation of S-phase cyclins, low levels and larger cytoplasmic localization of p27^kip1^ and ensuing sustained phosphorylation of pRb. Nevertheless transformed cells are unable to proceed with proliferation although maintaining a fairly high level of S-phase cells. Since glutamine is an important intermediate in purine and pyrimidine biosynthesis, glutamine exhaustion could deplete intracellular nucleotide pools, bringing in turn to a failure in the execution of a normal cell cycle [31], [36]. To test this hypothesis, we examined whether adding deoxyribonucleotides (using a concentration that has been shown to not interfere with proliferation of several cell lines *in vitro*) [37] could affect proliferation of normal and transformed cell lines. The presence of 10 µM dNTPs (dATP, dGTP, dTTP and dCTP) had no effect on growth of both normal and transformed cells in media supplemented with 4 mM glutamine (Figure S6, panels A and B) and on growth of normal cells in media supplemented with 0.5 mM glutamine (Figure 6A). On the contrary, addition of dNTPs sustained further growth of transformed cells, whether added once (at time 0 h) or twice (at time 0 h and 48 h) (compare squares, triangles and circles in Figure 6B). However, deoxyribonucleotides failed to revert the low glutamine-induced proliferation defect when added later, i.e. at 72 hours (data not shown). These results indicate that sustained supply of deoxyribonucleotides relieves glutamine dependence of proliferation in transformed cells.

To better analyze the G_1_ to S transition, cells grown in medium containing 4 mM glutamine were synchronized by serum starvation for 24 hours. As expected, transformed cells did not block in G_0_/G_1_ as effectively as normal cells (Figure 6 C through F), being 89% for normal and 78% for transformed, the percentage of cells with prereplicative DNA content as determined by bi-parametric BrdU/PI staining. The cell cycle block was released by adding 10% serum in media containing either 4 mM glutamine, 0.5 mM glutamine or 0.5 mM glutamine plus 10 µM dNTPs, and collected at indicated time points (8, 10, 12, 14, 16, 18 and 20 h). The time-course of transit through the cell cycle phases was then followed. Normal cells showed equal kinetics of re-entry into cell cycle regardless of glutamine concentration. The fraction of G_0_/G_1_ cells reached a minimum 14–16 hours after serum stimulation (panel E), while S-phase (panel G) and G_2_/M phase (panel I) peaked at 14 and 18 hours respectively. Transformed cells reproducibly anticipated their entry into S-phase as compared to normal cells (8 h *vs.* 10 h, panels G and H), regardless of glutamine concentration. The peak in S-phase (panel H) and G_2_/M phase (panel L) was also anticipated, but only in cells stimulated in media containing 4 mM glutamine (black bars) or 0.5 glutamine plus 10 µM dNTPs (white bars), but not in cells stimulated in media containing 0.5 mM glutamine (grey bars).

**Figure 7:**
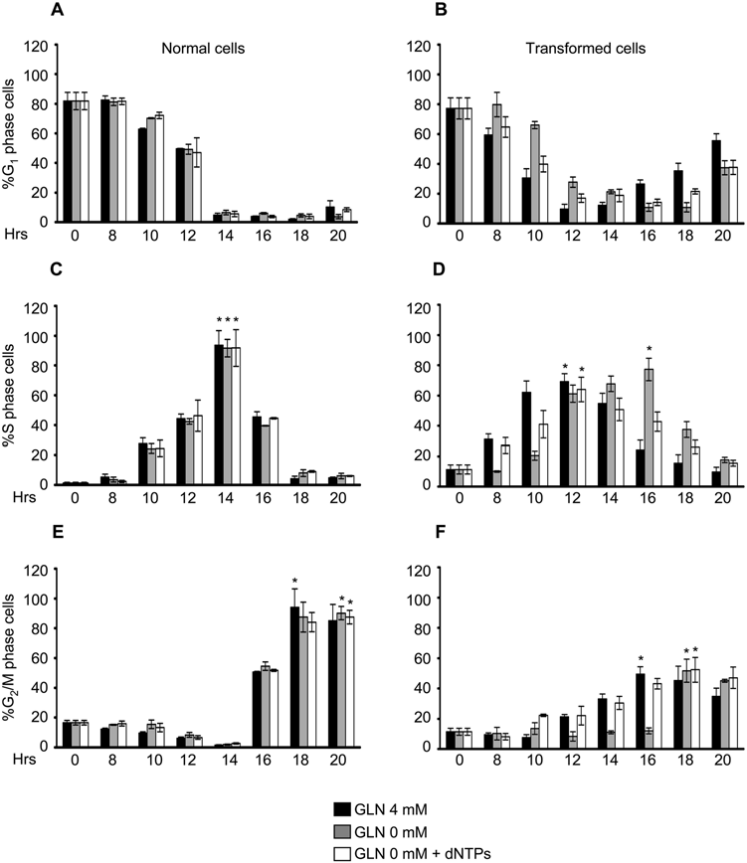
Cell cycle of synchronized normal and transformed cells released in 0 mM glutamine±dNTPs. Normal and transformed cells, synchronized by 24 hours of serum starvation, were released in a medium containing 10% serum, 4 mM or 0 mM glutamine, dNTPs and BrdU for continuous DNA labeling. At indicate time points the cells were collected and stained with propidium iodide and anti-BrdU specific antibody and analyzed by flow cytometry. The percentages of cells in G_1_ (panels A and B), S (panels C and D) and G_2_/M (panels E and F) phase of normal (left panels) and transformed (right panels) cells released in 4 mM and 0 mM glutamine alone or plus dNTPs obtained by bi-parametric analysis are represented. The asterisks indicate the S or G_2_/M phase peaks identified in both glutamine availability. The error bars correspond to standard deviations of triplicate analysis.

We next investigated whether nucleotide addition was effective also on cells released from a serum starvation block in complete absence of glutamine using the same protocol used in Figure 6. Cell cycle re-entry of normal cells was unaffected by release in 0 mM glutamine, regardless of the presence of nucleotides (Figure 7, panels A, C and E). In transformed cells, release in the absence of glutamine delayed S phase entrance of 2 hours (Figure 7, panels B and D) and delayed reaching of the S-phase peak of further 2 hours (Figure 7, panel D, asterisks). Addition of 10 µM dNTPs to transformed cells released in 0 glutamine (white bars) rescued the delay in S phase entrance, the delay in reaching the S phase peak, but did not rescue the delay in traversing the G_2_/M phase (panel F, compare black and white bars).

These data indicate that the major effect of glutamine limitation in transformed cells is to increase the length of S-phase, the peak of S-phase cells being reached 2 hours later in low glutamine (Figure 6 H, peaks indicated by asterisks) and 4 hours later in full absence of glutamine (Figure 7 D, peaks indicated by asterisks): this function of glutamine can be preserved by dNTPs. Complete glutamine depletion severely slows down transit through G_2_/M, regardless of dNTPs supply (Figure 7 F), indicating a distinct role of glutamine in this cell cycle phase.

### Response of selected signalling pathways to glutamine availability and effect of rapamycin treatment on proliferation ability in normal and transformed cells

In different transformed cell lines glutamine or other amino acids shortage has been reported to induce accumulation of cells in G_0_/G_1_ and/or an increase in doubling time and a decrease in maximal reached cell density [36], [38], [39]. Such anti-proliferative effects, most likely mediated by the TOR pathway [40], [41], have been associated either to a general decrease of mRNA translation and/or protein biosynthesis or to a specific decrease/increase of expression of both positive and negative regulators of cell cycle, as observed in cells deprived of arginine (decrease of Cdk4) [42], in cells deprived of hystidine (increase of p21^waf1^ and p27^kip1^) or methionine (increase of p21^waf1^) [43], [44]. In non-transformed cells, upstream activators such as PI3K/Akt and Ras/Raf/Erk and upstream inhibitors such as the phosphates PTEN and the AMP kinase properly control activation of TOR pathway. In transformed cells, the TOR pathway is deregulated through several pathways, among which a primary role is assigned to oncogenic Ras signalling, that is able to activate both positive regulators of TOR pathways (PI3K and ERK) [45], [46].

**Figure 8:**
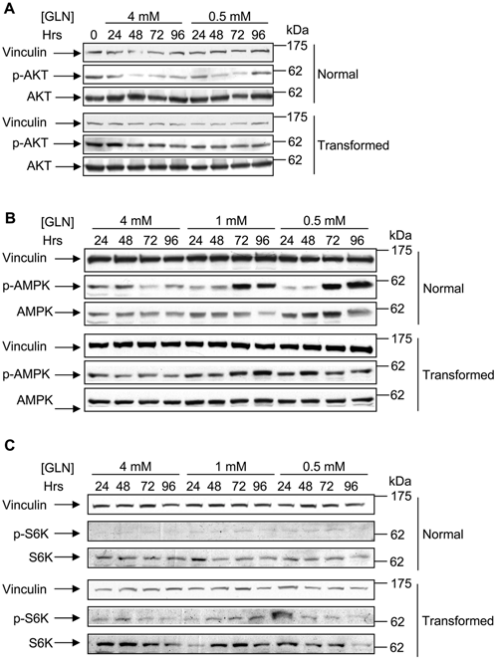
Activation of AKT, AMPK and TOR pathways in normal and transformed cells. (A) Time course expression and phosphorylation of the AKT protein in normal and transformed cell lines growing in media supplemented with different initial glutamine concentrations. For the protein expression analysis normal cells and transformed cells grown in media containing 4 mM glutamine and 0.5 mM glutamine, were collected at appropriate time points and 50 µg of proteins from the total cellular extract were subjected to SDS-PAGE followed by Western blotting with a specific anti-phosphorylated^Ser473^ AKT (p-AKT) antibody and an anti-AKT (AKT) antibody. (B) Time course expression and phosphorylation of the AMPKα protein in normal and transformed cell lines growing in media supplemented with different initial glutamine concentrations. For the protein expression analysis normal cells and transformed cells grown in media containing 4 mM glutamine, 1 mM glutamine and 0.5 mM glutamine, were collected at appropriate time points and 50 µg of proteins from the total cellular extract were subjected to SDS-PAGE followed by Western blotting with a specific anti-phosphorylated^Thr172^ AMPK (p-AMPK) antibody and an anti-AMPK (AMPK) antibody. (C) Time course expression and phosphorylation of the S6K protein in normal and transformed cell lines grown as described in B. Western blotting analysis has been performed by using a specific anti-phosphorylated^Thr389^ S6K (p-AKT) antibody and an anti-S6K (S6K) antibody. The panels are representative of a triplicate analysis (Akt and AMPK) or duplicate analysis (S6K).

To investigate the role of signal transduction in the response of normal and transformed cells to glutamine shortage, we analyzed the level of expression and activation of two main regulators of the TOR pathway, Akt and AMPK, as well as the level of expression and activation of a downstream TOR target p70 S6 kinase (S6K). As shown in Figure 8A, in normal cells, the activated form of Akt (probed with an anti-phosphoserine 473 antibody) decreases as a function of time and regardless initial glutamine concentration. In transformed cells the level of phosphorylated Akt is always very significant, being marginally higher at initial time points and not reduced as a function of time nor effected by glutamine concentration.

**Figure 9:**
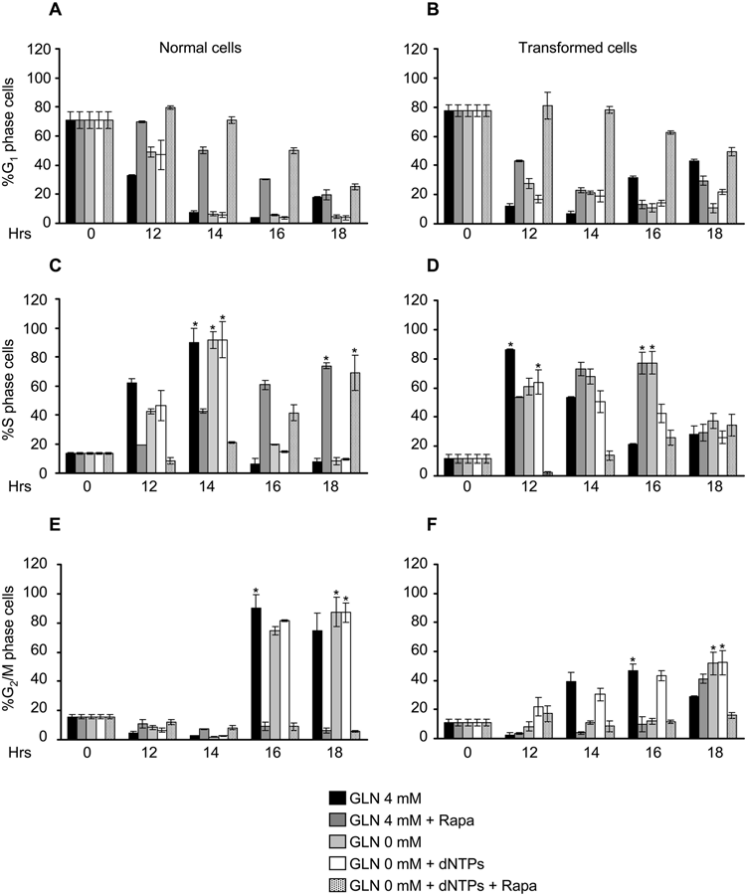
Analysis of cell cycle upon rapamycin treatment. Normal and transformed cells, synchronized by 24 hours of serum starvation, were released in a medium containing 10% serum, 4 mM glutamine±Rapamycin or 0 mM glutamine±Rapamycin and±dNTPs and BrdU for continuous DNA labeling. At indicate time points the cells were collected and stained with propidium iodide and anti-BrdU specific antibody and analyzed by flow cytometry. The percentages of cells in G_1_ (panels A and B), S (panels C and D) and G_2_/M (panels E and F) phase of normal (left panels) and transformed (right panels) cells, released in the mediums indicated above, and obtained by bi-parametric analysis are represented. The asterisks indicate the S or G_2_/M phase peaks identified in both glutamine availability. The error bars correspond to standard deviations of triplicate analysis.

As previously reported, TOR protein is also controlled through AMPK, which is activated by a drop in the ATP/ADP ratio with ensuing AMP accumulation that leads to a signaling bringing to different cellular responses as well as to cell cycle arrest [47]. Our Western blot experiments to analyze the AMPK activation status in cells grown in different glutamine availability, showed that in correlation with a drop of ATP levels in both cell lines, only in normal cells, in which the ATP decrease was greater (see Figure 5I, L and M), a reliable and glutamine-dependent increase of AMPK activation was observed (Figure 8B, 1 mM and 0.5 mM glutamine samples). Analysis of the p70 S6K phoshorylation (probed with an anti-phosphothreonine 389, which closely correlated with p70S6K activity that in turn is widely used biological readout for TOR pathway activation) indicated a greater activity as well as expression in all transformed cell samples as compared to normal cell samples. These results well correlate with several observations from other Authors, highlighting the importance of both Akt and S6K in oncogenic *ras* signaling [48]. To further explore the role of TOR pathway in the response of normal and transformed cells to glutamine shortage, the effects of rapamycin (known pharmacologic inhibitor of TOR protein activity) on ability of synchronized cells to enter in S phase was investigated. We examined whether adding rapamycin to synchronized normal and transformed cells, grown both in 4 mM or 0 mM glutamine plus dNTPs, we could interfere in particular with the execution of G_1_ to S transition in transformed cells also in presence of the dNTPs. As shown in Figure 9A, C and E, normal cell samples, treated with rapamycin, showed a delay to exit from the G_1_ arrest (panel A) and, thus a 4 hours delay to reach the S-phase peak (panel C, peaks indicated by asterisks) and consequently to execute the G_2_/M transition that was not observed in the interval of time choose for this specific experiment (panel E). Transformed cells, released in 4 mM glutamine plus rapamycin or 0 mM glutamine, showed a slower exit from G_1_ arrest and a 4 hours delay to reach the S-phase and the G_2_+M peaks (panel D and F, peaks indicated by asterisks) as compared to 4 mM sample and 0 mM plus dNTPs. Surprisingly, the sample released in 0 mM glutamine plus dNTPs and treated with rapamycin, was almost unable to exit from the G_1_ arrest and consequently to enter into S-phase. Indeed at later time point analyzed (18 h), were scored 50% and 34% of cells respectively in G_1_ and S phase. These findings were further confirmed by western blot analysis of cyclin D1 expression. Indeed in both normal and transformed cells treated with rapamycin the level of expression of this protein was lower than in untreated samples (data not shown).

Together such results indicate that in normal cells amino acid deprivation may participate in TOR signalling inhibition inducing a G_1_ arrest, (*i.e.* by reducing mRNA translation, protein synthesis, ATP levels and expression of cell cycle regulators), in *K-ras* transformed cells such control is deranged by activation of Akt and by strong inhibition of AMPK, that, acting in a concerted fashion, maintain also at low or absent glutamine availability a sizable activity of the TOR pathway, able to promote entrance into S phase. Indeed, TOR signaling inhibition in synchronized cells completely block the ability of transformed cells to enter in S phase also in presence of dNTPs.

## Discussion

A genetically defined experimental model in which the transformed phenotype is phenotypically reverted by expression of a dominant negative GEF protein that down regulates oncogenic *K-ras* [16], [17], has allowed us to show in this paper that glutamine deprivation induces abortive S-phase entrance in *K-ras* transformed cells while it does not participate to arrest normal and reverted cells in G_1_ phase.

**Figure 10:**
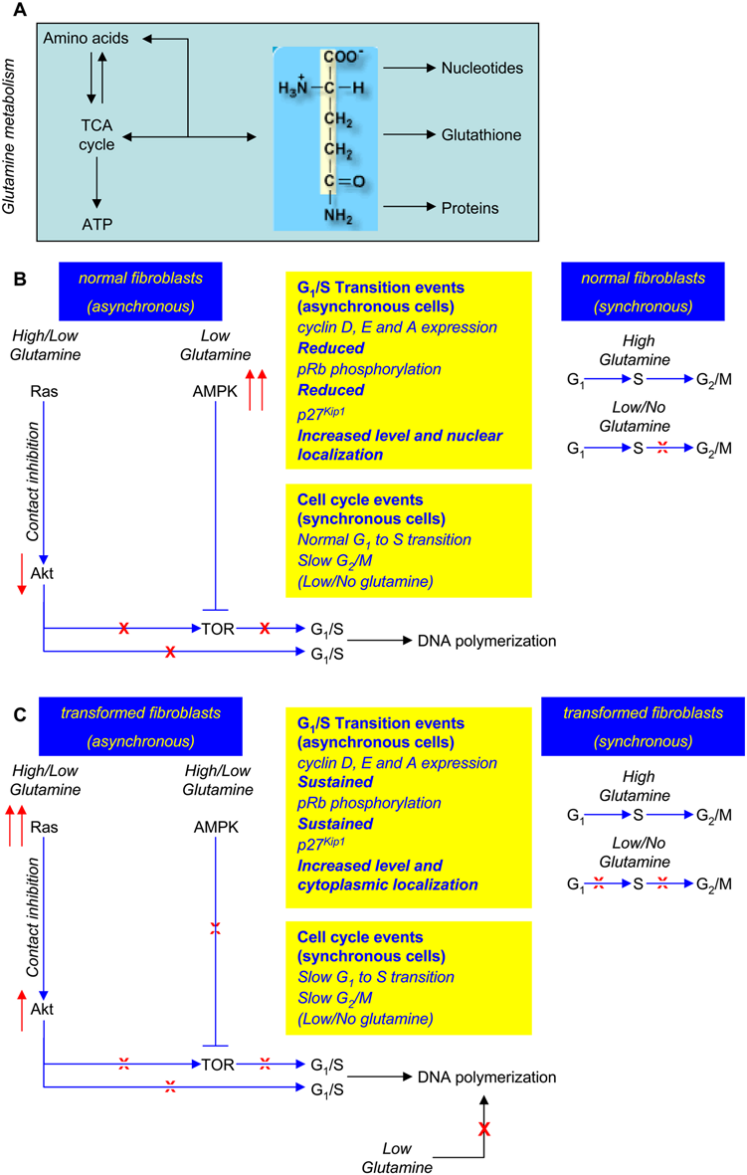
A model of the role of glutamine in controlling the G_1_/S transition. In the panels the following convention are used. Metabolic connections are indicated by black arrows. Blue lines indicate positive (arrowhead) or negative (blunt end) regulatory connections. Up- and down-regulation are indicated by red arrows pointing either upwards or downwards, respectively. A full block is indicated by a red X, a leaky block by a dotted X. (A) Some metabolic roles of glutamine are represented within a light blue area. (B) Summary of events originating from glutamine shortage in asynchronous and synchronous normal cells. Biological readouts of proteins involved in the G_1_ to S transition and the effects on cell cycle are reported. (C) Summary of events originating from glutamine shortage in asynchronous and synchronous *K-ras* transformed cells. Biological readouts of proteins involved in the G_1_ to S transition and the effects on cell cycle are reported.

The main results presented in this paper are recapitulated in Figure 10, in which the metabolic interconnections of glutamine (Figure 10A) and the effects that glutamine deprivation has on signaling pathways and molecular events of the G_1_ to S transition, both in normal/reverted or in *K-ras* transformed cells, are described (Figure 10B and C).

In asynchronous normal cells, contact inhibition, regardless of glutamine availability, brings to down-regulation of Akt that together with AMPK up-regulation, observed at low glutamine, will concur to TOR pathway inactivation. As a result, the expression of cyclin D, E and A is down regulated, pRb phosphorylation is strongly reduced, p27^kip1^ level is increased and its localization becomes preferentially nuclear, establishing therefore a condition that bring to a G_1_- cell cycle arrest (Figure 10B). In synchronous normal cells, glutamine shortage slows the G_2_/M transition, indicating a possible role of glutamine in such cell cycle phase.

In *K-ras* transformed cells, in which the level of activated Ras-GTP is very high [17] and the contact inhibition is less efficient [17], the deprivation of glutamine affects Akt and AMPK in a way opposite to that observed in normal cells, leaving the TOR pathway at least partially activated (Figure 10C). This event allows sizable expression of cyclin D (at least until 72 hours), E and A, sustained pRb phosphorylation, decreased p27^kip1^ and its preferential cytoplasmic localization, conditions that, taken together, promote entrance into S phase (Figure 10C). In synchronous transformed cells, glutamine shortage slows both the G_1_ to S transition or the G_2_/M transition.

But why in low glutamine is the S phase of *K-ras* transformed cells abortive? Deprivation of glutamine reduces also availability of nucleotides that leads to a small decrease in RNA accumulation compared to growth in high glutamine (at least until the 72 hours, Fig. 5B) and a more dramatic effect on DNA synthesis (Figure 1F, S1D). The notion that depletion of deoxyribonucleotides is the major effect of glutamine deprivation that leads to reduced proliferation of transformed fibroblasts, is confirmed by the fact that the proliferation defect of transformed cells is rescued by adding these precursors of DNA polymerization to low glutamine medium (Figure 6B).

When the low amount of glutamine is still compatible with cell cycle progression, as in serum starved cells substantially synchronized in G_1_ (Figure 6), in transformed cells low-glutamine medium causes a 2 hours delay in entering into S-phase after serum re-addition. The same delay is transmitted to the G_2_/M transition, indicating that no major compensatory mechanism on cell cycle timing between completion of S-phase and cell division are operative. This effect on cell cycle timing is worsened by complete absence of glutamine (Figure 7), in which 4 hours delay in entering into S-phase and in the execution of G_2_/M transition have been observed. These data strongly indicate that the effect of glutamine limitation in transformed cells is first to slow down the S-phase traverse, then, when a more severe limitation is established, to stuck a large fraction of the cells population in S phase.

Studies performed in several cellular models have shown that availability of dNTPs and therefore the regulation of their synthesis, plays a critical role in DNA replication [49]. Indeed expression of dNTP synthetic enzymes is cell cycle-regulated, with enhanced expression at the onset of S-phase [50], [51]. Additionally, dNTP levels vary within S-phase of the cell cycle [52] thereby changing the rate of DNA replication during S-phase [53]. Even subtle changes in the levels of dNTPs may have a sizable effect on DNA replication [54]. Nucleotide depletion, obtained through chemical inhibition of their biosynthesis, may induce either G_1_ or S-phase arrest or slow down the overall progression of S-phase [55], [56]. Addition of a mix of 10 µM deoxyribonucleotides reverted effects on both asynchronous and asynchronous cultures indicating that the earlier and major rate-limiting step dependent on glutamine-deprivation pathway is connected with nucleotide biosynthesis. Nevertheless, such connections appear to be lost both in asynchronous and synchronous cells when a complete limitation of glutamine in the culture medium occurs. Indeed at later time points of low glutamine proliferation curve of transformed cells (72–96 h), corresponding to almost a complete depletion of glutamine in the culture medium (Figure S2B), dNTPs are not more able to restore the proliferation (Figure 6B) indicating that in such condition other cellular responses are activated by glutamine depletion. Furthermore in transformed synchronous cells, released in complete glutamine depleted medium, dNTPs are able to restore the S-phase transition but not the proper time of execution of G_2_/M phase (Figure 7F). Indeed such cell cycle phase was delayed as well as observed in the sample without glutamine and dNTPs. Other Authors have observed this result in different cell lines. For instance, Abcouwer et al. [57], showed that reduction of glutamine availability in the growth medium of several breast cancer cells might induce GADD45 and GADD153 expression by mRNA stabilization. GADD45 overexpression has been shown to induce G_2_/M cell cycle arrest [58] following several environmental stresses. Moreover, Drogat et al. [38], showed that in A549/8 carcinoma cells glutamine deprivation decreased the number of cells in the G_1_ phase, transiently increased that of cells in the S phase, and induced a stable increase in cell number in the G_2_/M phase. These findings may explain the delay to reach the G_2_/M peak observed in both normal and transformed cells released in low or complete absence of glutamine (Figure 6 and 7).

In cells exhibiting high metabolic rates, such as rapidly dividing cancer cells grown *in vitro*, glutamine, being the most readily available amino acid used as energy source, may became the major source of energy to sustain protein and nucleic acid synthesis [11], especially when glucose levels are low and energy demand is high. A correlation between glutamine depletion and decreased level of Krebs cycle intermediates in *myc*-transformed cells has been identified [35].

In our system metabolic readouts of transformed cells - including ATP, RNA and protein levels - are quite close to those found in normal and reverted cells. Addition of Krebs cycle intermediates has no effect on *K-ras* transformed fibroblasts (Figure 5 and S5) thereby suggesting that proliferation arrest may be independent on Krebs cycle intermediates depletion and energy shortage. On the contrary, we showed that ATP drops in the same cell lines grown under limiting glucose availability, linked to inability of these cells to properly activate mitochondrial respiration [14], [15], eventually leading to cell death. Such partial derangement of mitochondrial function is further confirmed in the present report, since we show that *K-ras* transformed cells grown in a medium in which glucose was completely replaced by 1 mM pyruvate and 4 mM glutamine, substrates used essentially for Krebs cycle and oxidative respiration, grow poorly (Figure S7) and eventually die (data not shown).

In conclusion, glutamine shortage in *K-ras* transformed cells limits proliferation by inducing abortive S-phase entrance, while glucose shortage in the same system enhanced cell death [14], [15]. The differential effects of glutamine and glucose on cell viability are not a property of the transformed phenotype *per se*, but rather depend on the specific pathway being activated in transformation. For instance, *myc*-overexpressing cells die under complete glutamine depletion in a *myc*-dependent way and not under glucose depletion [35]. In the same experimental conditions, complete glutamine depletion, cell death of *K-ras* transformed cells die is marginal compared to that observed in *myc*-overexpressing cells (data not shown). These different responses of transformed cells to nutritional stress should be taken into account when designing anti-cancer therapies that aim to exploit metabolic differences between normal and transformed cells.

## Materials and Methods

### Cell culture

Mouse embryonic fibroblast NIH3T3 cells (CRL-1658; American Type Culture Collection) and a *K-Ras* transformed NIH3T3-derived cell line, 226.4.1 [59], were routinely grown in Dulbecco's modified Eagle's medium containing 10% newborn calf serum, 4 mM L-glutamine, 100 U/ml penicillin and 100 mg/ml streptomycin (normal growth medium) (all from Invitrogen, Carlsbad, CA, USA) at 37°C in a humidified atmosphere of 5% CO_2_. The reverted cell line, stably and constitutively expressing the dominant negative mutant Cdc25^MmW1056E^ (GEF-DN) [17] was maintained in normal growth medium supplemented with 0.7 mg/ml geneticin (G418; Sigma-Aldrich Inc., St. Louis, MO, USA). Cells were passaged using trypsin-ethylenediaminetetraacetic acid (EDTA) (Invitrogen) and maintained in culture for 48 h before manipulation.

### Cell treatments

To verify cell response to glutamine, cells were plated at 3000 cells/cm^2^ in normal growth medium (4 mM Glutamine). 18 h after seeding, cells were washed 2× with phosphate-buffered saline (PBS) and incubated in media with different glutamine (GLN) concentrations (4 mM, 1 mM and 0.5 mM glutamine). When required, 2 mM pyruvate (Sigma), 2 mM malate (Sigma), 10 µM dNTPs (Rovalab), and 20 nM rapamycin (ALEXIS Biochemicals), were added as indicated in the text.

### Cell synchronization

Cells were plated at 5500 cells/cm^2^ in normal growth medium. 18 h after the seeding, cells were washed 2× with PBS and synchronized by 24 h of serum starvation with Dulbecco's modified Eagle's medium containing 4 mM L-glutamine, 100 U/ml penicillin and 100 µg/ml streptomycin (Invitrogen), 6 ng/ml sodium selenite and 6 µg/ml transferrin (Sigma). Stimulation of quiescent cells was performed by adding 10% newborn calf serum in medium with the appropriate glutamine (and dNTPs, when required) concentration. DNA was labelled with 33 µM 5-Bromodeoxyuridine.

### Flow cytometric analysis

The distribution of cells at specific cell cycle phases was evaluated by flow cytometry. Cells were trypsinized, washed with PBS and fixed in 75% ethanol at 4°C. The cells were washed in PBS for ethanol removal and incubated for 30 min in 0.25% Triton X-100 and HCl 2N. Subsequently, each sample was stained with an anti-BrdU primary antibody (Becton-Dickinson) for 1 h, and probed with Alexa Fluor 488 donkey anti-mouse IgG (Molecular Probes/Invitrogen) to identify S-phase. In addition, the same samples were stained with propidium iodide (Sigma) and analyzed by FACS (FACScan, Becton-Dickinson), using the Cell Quest software (BD Bioscience). Data analysis was performed with WinMDI software.

### Glutamine assay

Glutamine variation in supernatants of normal and transformed fibroblasts was determined by using a spectrophotometric glutamine/glutamate enzyme assay kit (Sigma) based on enzymatic deamination of L-glutamine and dehydrogenation of L-glutamate with conversion of NAD^+^ to NADH. The assay was performed as specified by manufacturer datasheet and it is specific for glutamine and does not cross-react with other amino acids or ammonia. To calculate the quantity of glutamine a linear regression analysis of the standard curve was performed.

### Immunofluorescence microscopy

Cells were grown on coverslip previously treated with 0.2% gelatine. The cells were washed 3× with PBS and fixed in 4% paraformaldehyde in PBS for 10 min. Subsequently, were washed 3× with PBS, permeabilized with 0.1% Triton X-100 for 10 min, blocked with PBS+10% goat serum for 30 min, probed with primary antibody p27^kip1^ (1∶100) (Santa Cruz Biotechnology CA, USA) in PBS+10% goat serum for 1 h at room temperature, washed 3× with PBS, probed with fluorescence-labeled secondary antibody Alexa Fluor 594 goat anti-rabbit IgG (1∶400) (Molecular Probes/Invitrogen) in PBS+10% goat serum for 30 min at room temperature, washed 3× with PBS, stained with DAPI for 2 min (1∶500) (Sigma-Aldrich Inc), and then mounted in DABCO (Sigma-Aldrich Inc). For BrdU (Sigma-Aldrich Inc.) labeling, cells were fixed, permeabilized and blocked as described above. After the cells were incubate with an anti-BrdU monoclonal antibody (1∶10) (Becton-Dickinson), MgCl_2_ (3 mM) and DNasi I (Invitrogen) 100 U/ml for 1 h at RT. Subsequently, the cells were washed 3× with PBS and incubated with fluorescence-labeled secondary antibody Alexa Fluor 488 donkey anti-mouse IgG (1∶100) (Molecular Probes/Invitrogen) in PBS+10% goat serum for 30 min at room temperature, washed 3× with PBS, stained with DAPI for 2 min (1∶500) and then mounted in DABCO.

### Microscopy

The cover glasses, mounted in DABCO, were analyzed under a Nikon ECLIPSE 90i fluorescence microscope equipped with a b/w CCD camera (Hamamatsu-CoolSNAP, Hamamatsu Corporation Japan), using Plan Apo objective (40× dry and 60× oil; numerical aperture 0.75 and 1.4 respectively). The images were acquired using the imaging software Metamorph 7, then processed in Adobe Photoshop 7.0.1 with adjustments of brightness and contrast. The quantitative analysis image was performed using the imaging software Image J. The relative distribution of p27^kip1^ protein between the two compartments nucleo/cytoplasm, was calculated by measuring the pixel average signal both in the nucleus, cytoplasm and nucleo/cytoplasm compartments. At least 200 cells, together with negative controls (no primary antibody), were randomly selected. In order to exclude the background of staining and to select the positive stained parts of the cells for measurement, the images from the various samples were processed at the same threshold and then measured.

### Immunoblot analysis

Cells were lised in a buffer containing 150 mM NaCl, 0.5% NP-40, 1% glycerol, 50 mM HEPES (pH 7.5), 5 mM ethyleneglycol tetracetate (EGTA), 1 mM phenylmethylsulphonyl (PMSF), 50 mM NaF and a cocktail of protease inhibitors (Roche). After incubation for 30 min on ice, the extracts were centrifuged at 13.200 r.p.m. for 20 min. Protein concentration of supernatant was measured by the Bradford procedure (Bio-Rad Laboratories, Richmond, CA, USA), using bovine serum albumin as a standard. These cellular extracts were electrophoresed in sodium dodecylsulfate (SDS) polyacrylamide gels. After electrophoresis, the proteins were transferred to nitrocellulose membrane by electroblotting and incubated with antibodies over night.

The antibodies used were monoclonal or polyclonal antibodies against cyclin D1, cyclin E, cyclin A, p27^Kip1^, Cdk2, Cdk4 (all from Santa Cruz Biotechnology), phosphoRb-795, p70 S6 kinase, phospho-p70 S6 kinase and vinculine (all from Cell Signalling). Subsequently, the membranes were incubated with a peroxidase-coupled secondary antibody (Amersham, Othelfingen, CH) for 30 min at room temperature. The reaction was visualized with ECL (Amersham) followed by exposure to an x-ray film. Protein expression levels were quantified by densitometric evaluation of antibody specific bands on scanned x-ray films by using the imaging software Image J.

### RNA extraction and analysis

Total RNA was isolated from normal and transformed cell lines using TRIzol reagent (Invitrogen). RNA purity and integrity were checked by direct observation loading 1 µg of total RNA on 1% agarose gel. Total RNA had a 28S∶18S rRNA ratio of at least 2.0. Amount estimation was done by spectrophotometer analysis.

## Supporting Information

Figure S1 Analysis of glutamine concentration, along a time course of 96 h, of normal (blue diamond) and transformed (red square) cells plated in 4 mM (A) and 0.5 mM (B) initial glutamine availability.
(1.25 MB TIF)

Figure S2 Proliferation and cell cycle analysis of lower density plated cells.
(8.16 MB TIF)

Figure S3 Cdk2 and Cdk4 proteins expression is more stable in transformed cells.
(1.56 MB TIF)

Figure S4 p27kip1 localization in BrdU positive cells and p21waf1 expression in normal and transformed cells.
(2.65 MB TIF)

Figure S5 Glutamine availability effect on total cellular proteins as detected by Gel-Code Blue Comassie staining.
(4.43 MB TIF)

Figure S6 Proliferation ability and time of re-entry upon serum starvation and release in cells grown in 4 mM glutamine.
(2.68 MB TIF)

Figure S7 Glucose depletion induces a strong cell proliferation arrest in K-ras transformed mouse fibroblasts.
(1.15 MB TIF)

## References

[1] Deberardinis RJ, Lum JJ, Hatzivassiliou G, Thompson CB (2008). The biology of cancer: metabolic reprogramming fuels cell growth and proliferation.. Cell Metab.

[2] Ramanathan A, Wang C, Schreiber SL (2005). Perturbational profiling of a cell-line model of tumorigenesis by using metabolic measurements.. Proc Natl Acad Sci U S A.

[3] Warburg O (1956). On the origin of cancer cells.. Science.

[4] Mathupala SP, Rempe A, Pedersen PL (1997). Aberrant glycolytic metabolism of cancer cells: a remarkable coordination of genetic, transcriptional, post-translational, and mutational events that lead to a critical role for type II hexokinase.. J Bioenerg Biomembr.

[5] Mazurek S, Eigenbrodt E (2003). The tumor metabolome.. Anticancer Res.

[6] McFate T, Mohyeldin A, Lu H, Thakar J, Henriques J (2008). Pyruvate dehydrogenase complex activity controls metabolic and malignant phenotype in cancer cells.. J Biol Chem.

[7] Carew JS, Huang P (2002). Mitochondrial defects in cancer.. Mol Cancer.

[8] Modica-Napolitano JS, Singh KK (2004). Mitochondrial dysfunction in cancer.. Mitochondrion.

[9] Wallace DC (2005). A mitochondrial paradigm of metabolic and degenerative diseases, aging, and cancer: a dawn for evolutionary medicine.. Annu Rev Genet.

[10] Gatenby RA, Gillies RJ (2004). Why do cancers have high aerobic glycolysis?. Nat Rev Cancer.

[11] Curi R, Lagranha CJ, Doi SQ, Sellitti DF, Procopio J (2005). Molecular mechanisms of glutamine action.. J Cell Physiol.

[12] Kahn S, Yamamoto F, Almoguera C, Winter E, Forrester K (1987). The c-K-ras gene and human cancer (review).. Anticancer Res.

[13] Yamamoto F, Perucho M (1984). Activation of a human c-K-ras oncogene.. Nucleic Acids Res.

[14] Chiaradonna F, Gaglio D, Vanoni M, Alberghina L (2006). Expression of transforming K-Ras oncogene affects mitochondrial function and morphology in mouse fibroblasts.. Biochim Biophys Acta.

[15] Chiaradonna F, Sacco E, Manzoni R, Giorgio M, Vanoni M (2006). Ras-dependent carbon metabolism and transformation in mouse fibroblasts.. Oncogene.

[16] Vanoni M, Bertini R, Sacco E, Fontanella L, Rieppi M (1999). Characterization and properties of dominant-negative mutants of the ras-specific guanine nucleotide exchange factor CDC25(Mm).. J Biol Chem.

[17] Bossu' P, Vanoni M, Wanke V, Cesaroni MP, Tropea F (2000). A dominant negative RAS-specific guanine nucleotide exchange factor reverses neoplastic phenotype in K-ras transformed mouse fibroblasts.. Oncogene.

[18] Filmus J, Robles AI, Shi W, Wong MJ, Colombo LL (1994). Induction of cyclin D1 overexpression by activated ras.. Oncogene.

[19] Albanese C, Johnson J, Watanabe G, Eklund N, Vu D (1995). Transforming p21ras mutants and c-Ets-2 activate the cyclin D1 promoter through distinguishable regions.. J Biol Chem.

[20] Harbour JW, Dean DC (2000). The Rb/E2F pathway: expanding roles and emerging paradigms.. Genes Dev.

[21] Cheng M, Olivier P, Diehl JA, Fero M, Roussel MF (1999). The p21(Cip1) and p27(Kip1) CDK ‘inhibitors’ are essential activators of cyclin D-dependent kinases in murine fibroblasts.. Embo J.

[22] Chang F, McCubrey JA (2001). P21(Cip1) induced by Raf is associated with increased Cdk4 activity in hematopoietic cells.. Oncogene.

[23] Liang J, Zubovitz J, Petrocelli T, Kotchetkov R, Connor MK (2002). PKB/Akt phosphorylates p27, impairs nuclear import of p27 and opposes p27-mediated G1 arrest.. Nat Med.

[24] Shin I, Yakes FM, Rojo F, Shin NY, Bakin AV (2002). PKB/Akt mediates cell-cycle progression by phosphorylation of p27(Kip1) at threonine 157 and modulation of its cellular localization.. Nat Med.

[25] Viglietto G, Motti ML, Bruni P, Melillo RM, D'Alessio A (2002). Cytoplasmic relocalization and inhibition of the cyclin-dependent kinase inhibitor p27(Kip1) by PKB/Akt-mediated phosphorylation in breast cancer.. Nat Med.

[26] Everson WV, Flaim KE, Susco DM, Kimball SR, Jefferson LS (1989). Effect of amino acid deprivation on initiation of protein synthesis in rat hepatocytes.. Am J Physiol.

[27] Kimball SR (2001). Regulation of translation initiation by amino acids in eukaryotic cells.. Prog Mol Subcell Biol.

[28] Le Bacquer O, Nazih H, Blottiere H, Meynial-Denis D, Laboisse C (2001). Effects of glutamine deprivation on protein synthesis in a model of human enterocytes in culture.. Am J Physiol Gastrointest Liver Physiol.

[29] Higashiguchi T, Hasselgren PO, Wagner K, Fischer JE (1993). Effect of glutamine on protein synthesis in isolated intestinal epithelial cells.. JPEN J Parenter Enteral Nutr.

[30] MacLennan PA, Brown RA, Rennie MJ (1987). A positive relationship between protein synthetic rate and intracellular glutamine concentration in perfused rat skeletal muscle.. FEBS Lett.

[31] Boza JJ, Moennoz D, Bournot CE, Blum S, Zbinden I (2000). Role of glutamine on the de novo purine nucleotide synthesis in Caco-2 cells.. Eur J Nutr.

[32] Baggetto LG (1992). Deviant energetic metabolism of glycolytic cancer cells.. Biochimie.

[33] Zielke HR, Zielke CL, Ozand PT (1984). Glutamine: a major energy source for cultured mammalian cells.. Fed Proc.

[34] DeBerardinis RJ, Mancuso A, Daikhin E, Nissim I, Yudkoff M (2007). Beyond aerobic glycolysis: transformed cells can engage in glutamine metabolism that exceeds the requirement for protein and nucleotide synthesis.. Proc Natl Acad Sci U S A.

[35] Yuneva M, Zamboni N, Oefner P, Sachidanandam R, Lazebnik Y (2007). Deficiency in glutamine but not glucose induces MYC-dependent apoptosis in human cells.. J Cell Biol.

[36] Wasa M, Bode BP, Abcouwer SF, Collins CL, Tanabe KK (1996). Glutamine as a regulator of DNA and protein biosynthesis in human solid tumor cell lines.. Ann Surg.

[37] Rathbone MP, Middlemiss PJ, Gysbers JW, DeForge S, Costello P (1992). Purine nucleosides and nucleotides stimulate proliferation of a wide range of cell types.. In Vitro Cell Dev Biol.

[38] Drogat B, Bouchecareilh M, North S, Petibois C, Deleris G (2007). Acute L-glutamine deprivation compromises VEGF-a up-regulation in A549/8 human carcinoma cells.. J Cell Physiol.

[39] Turowski GA, Rashid Z, Hong F, Madri JA, Basson MD (1994). Glutamine modulates phenotype and stimulates proliferation in human colon cancer cell lines.. Cancer Res.

[40] Arsham AM, Neufeld TP (2006). Thinking globally and acting locally with TOR.. Curr Opin Cell Biol.

[41] Edinger AL (2007). Controlling cell growth and survival through regulated nutrient transporter expression.. Biochem J.

[42] Lamb J, Wheatley DN (2000). Single amino acid (arginine) deprivation induces G1 arrest associated with inhibition of cdk4 expression in cultured human diploid fibroblasts.. Exp Cell Res.

[43] Leung-Pineda V, Pan Y, Chen H, Kilberg MS (2004). Induction of p21 and p27 expression by amino acid deprivation of HepG2 human hepatoma cells involves mRNA stabilization.. Biochem J.

[44] Kokkinakis DM, Liu X, Neuner RD (2005). Modulation of cell cycle and gene expression in pancreatic tumor cell lines by methionine deprivation (methionine stress): implications to the therapy of pancreatic adenocarcinoma.. Mol Cancer Ther.

[45] Zhao JJ, Cheng H, Jia S, Wang L, Gjoerup OV (2006). The p110alpha isoform of PI3K is essential for proper growth factor signaling and oncogenic transformation.. Proc Natl Acad Sci U S A.

[46] Conde E, Angulo B, Tang M, Morente M, Torres-Lanzas J (2006). Molecular context of the EGFR mutations: evidence for the activation of mTOR/S6K signaling.. Clin Cancer Res.

[47] Zhuang Y, Miskimins WK (2008). Cell cycle arrest in Metformin treated breast cancer cells involves activation of AMPK, down-regulation of cyclin D1, and requires p27Kip1 or p21Cip1.. J Mol Signal.

[48] Friday BB, Adjei AA (2005). K-ras as a target for cancer therapy.. Biochim Biophys Acta.

[49] Eriksson S, Skog S, Tribukait B, Jaderberg K (1984). Deoxyribonucleoside triphosphate metabolism and the mammalian cell cycle. Effects of thymidine on wild-type and dCMP deaminase-deficient mouse S49 T-lymphoma cells.. Exp Cell Res.

[50] Sherley JL, Kelly TJ (1988). Regulation of human thymidine kinase during the cell cycle.. J Biol Chem.

[51] Eriksson S, Graslund A, Skog S, Thelander L, Tribukait B (1984). Cell cycle-dependent regulation of mammalian ribonucleotide reductase. The S phase-correlated increase in subunit M2 is regulated by de novo protein synthesis.. J Biol Chem.

[52] Eriksson S, Groppi V, Ullman B, Martin DW (1984). Cell-cycle dependent variation in the levels of deoxyribonucleoside triphosphate in mouse T-lymphoma cells.. Adv Exp Med Biol.

[53] Collins JM (1978). Rates of DNA synthesis during the S-phase of HeLa cells.. J Biol Chem.

[54] Mathews CK (2006). DNA precursor metabolism and genomic stability.. Faseb J.

[55] Agarwal ML, Agarwal A, Taylor WR, Chernova O, Sharma Y (1998). A p53-dependent S-phase checkpoint helps to protect cells from DNA damage in response to starvation for pyrimidine nucleotides.. Proc Natl Acad Sci U S A.

[56] Agarwal MK, Hastak K, Jackson MW, Breit SN, Stark GR (2006). Macrophage inhibitory cytokine 1 mediates a p53-dependent protective arrest in S phase in response to starvation for DNA precursors.. Proc Natl Acad Sci U S A.

[57] Abcouwer SF, Schwarz C, Meguid RA (1999). Glutamine deprivation induces the expression of GADD45 and GADD153 primarily by mRNA stabilization.. J Biol Chem.

[58] Wang XW, Zhan Q, Coursen JD, Khan MA, Kontny HU, Yu L (1999). GADD45 induction of a G2/M cell cycle checkpoint.. Proc Natl Acad Sci U S A.

[59] Pulciani S, Santos E, Long LK, Sorrentino V, Barbacid M (1985). ras gene Amplification and malignant transformation.. Mol Cell Biol.

