# Supplementary figures and images for: Glutamine Deprivation Induces Abortive S-Phase Rescued by Deoxyribonucleotides in K-Ras Transformed Fibroblasts
Source: PLoS One. 2009 Mar 5;4(3):e4715. doi: 10.1371/journal.pone.0004715 (PMC2650790; doi:10.1371/journal.pone.0004715)

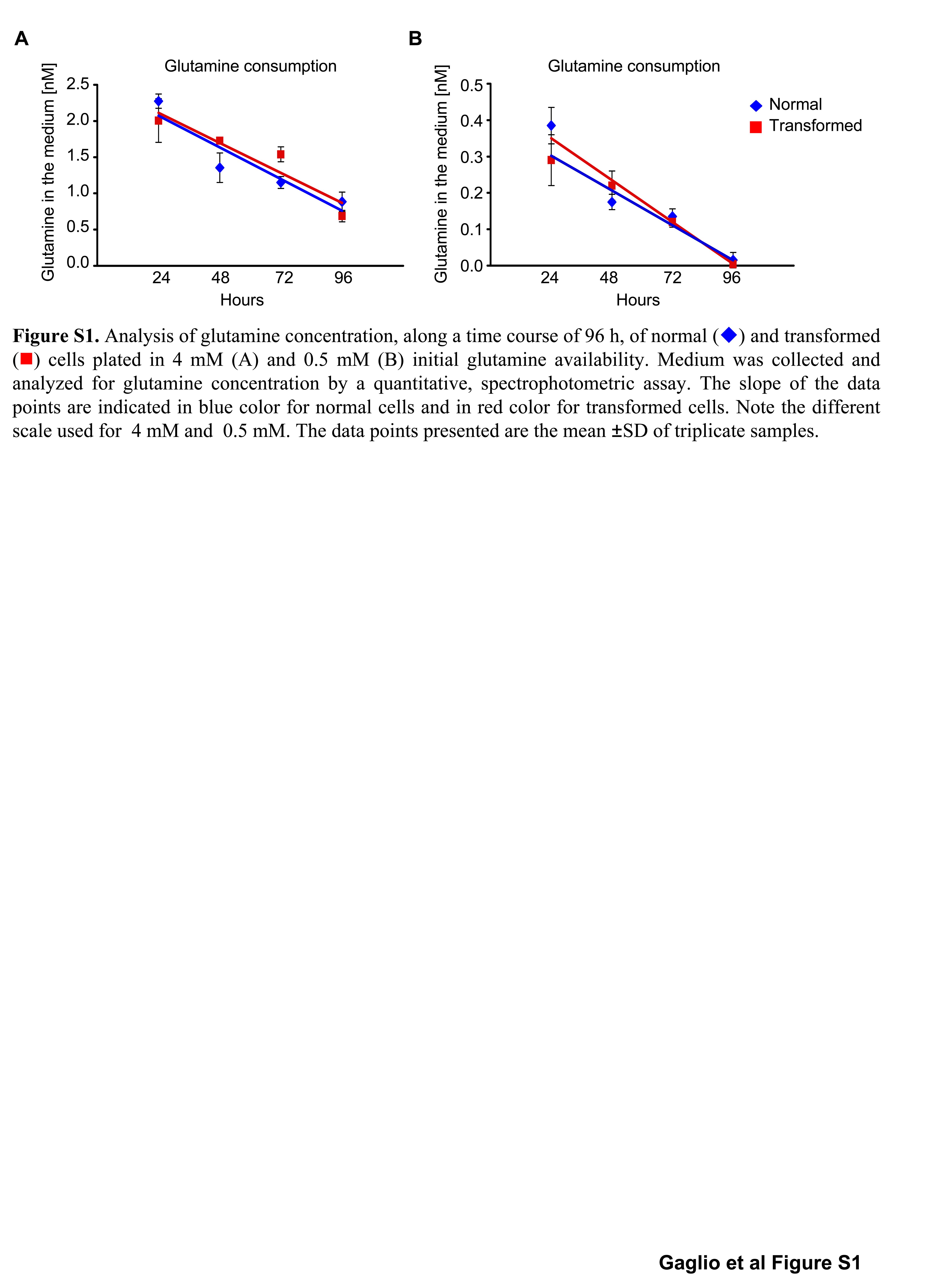

Supplement: Figure S1 — Analysis of glutamine concentration, along a time course of 96 h, of normal (blue diamond) and transformed (red square) cells plated in 4 mM (A) and 0.5 mM (B) initial glutamine availability. (1.25 MB TIF) [file pone.0004715.s001.tif]

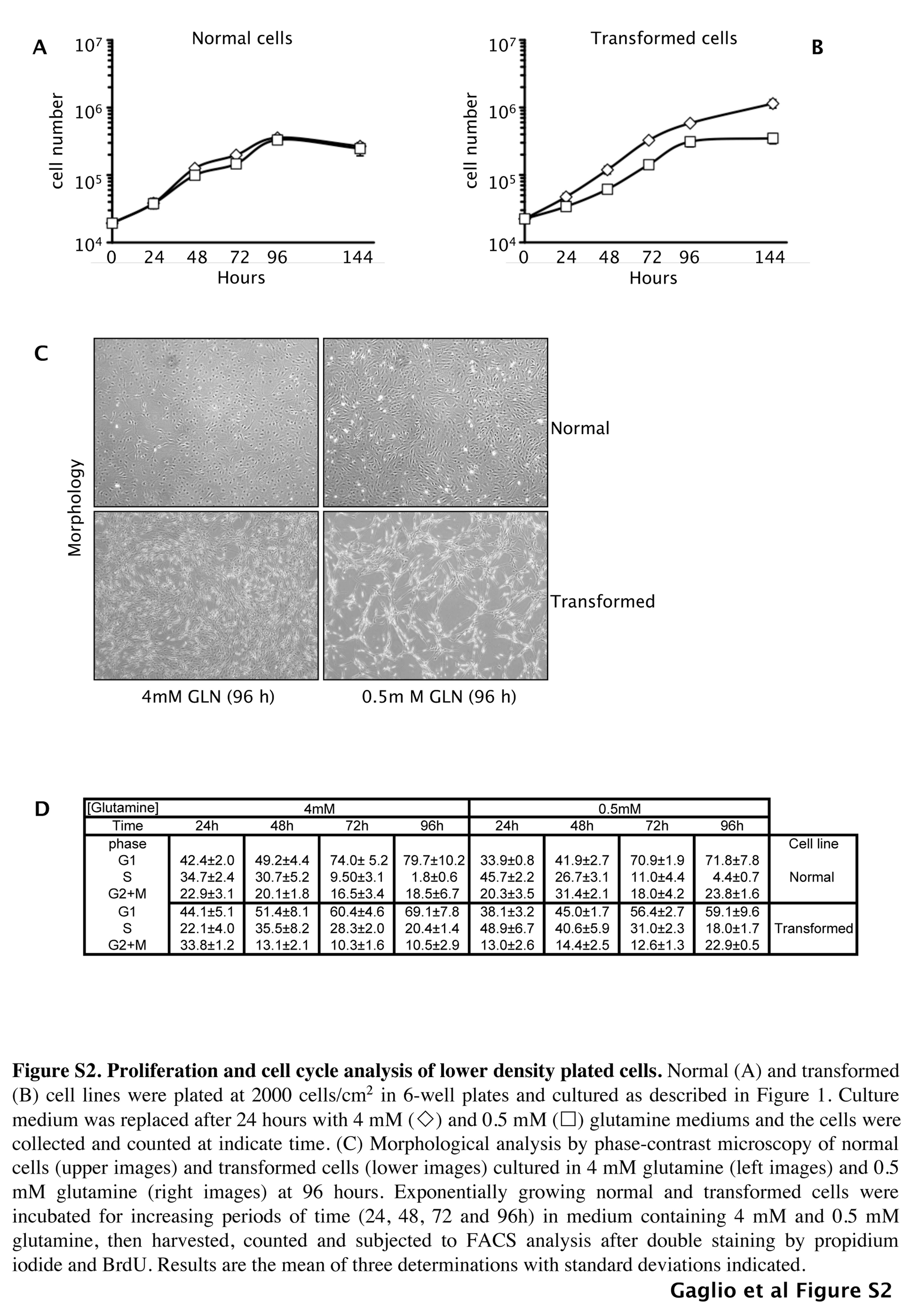

Supplement: Figure S2 — Proliferation and cell cycle analysis of lower density plated cells. (8.16 MB TIF) [file pone.0004715.s002.tif]

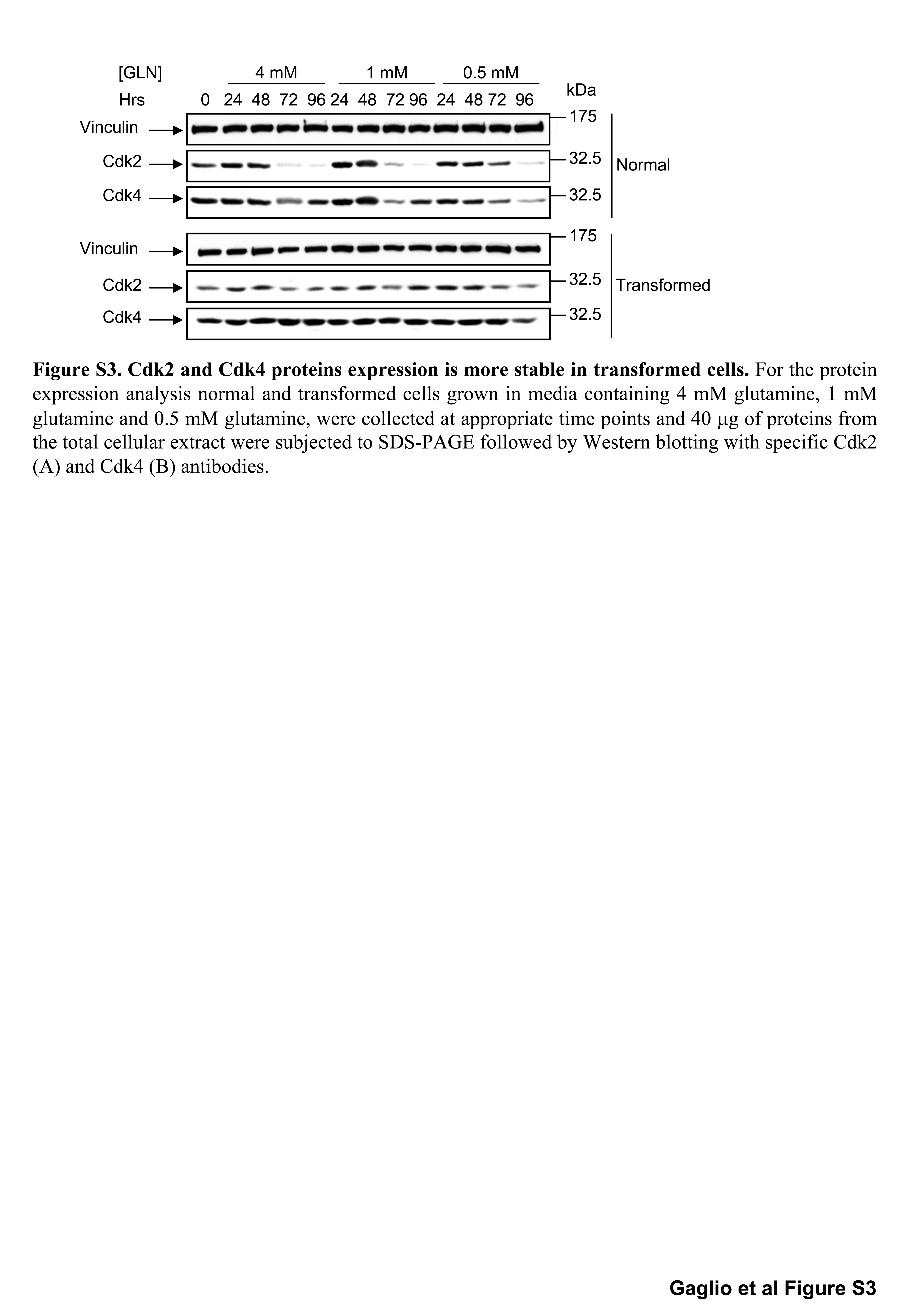

Supplement: Figure S3 — Cdk2 and Cdk4 proteins expression is more stable in transformed cells. (1.56 MB TIF) [file pone.0004715.s003.tif]

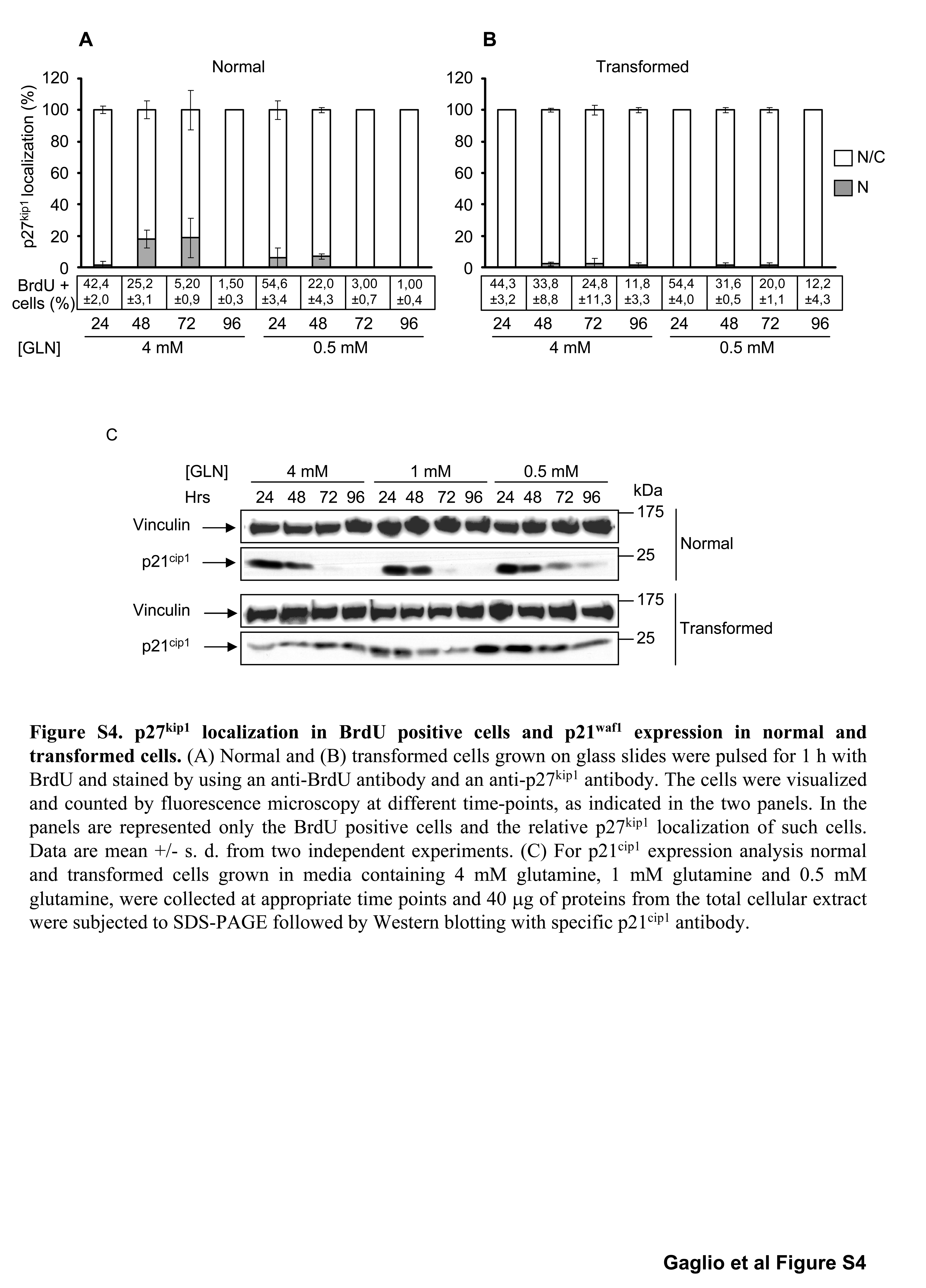

Supplement: Figure S4 — p27kip1 localization in BrdU positive cells and p21waf1 expression in normal and transformed cells. (2.65 MB TIF) [file pone.0004715.s004.tif]

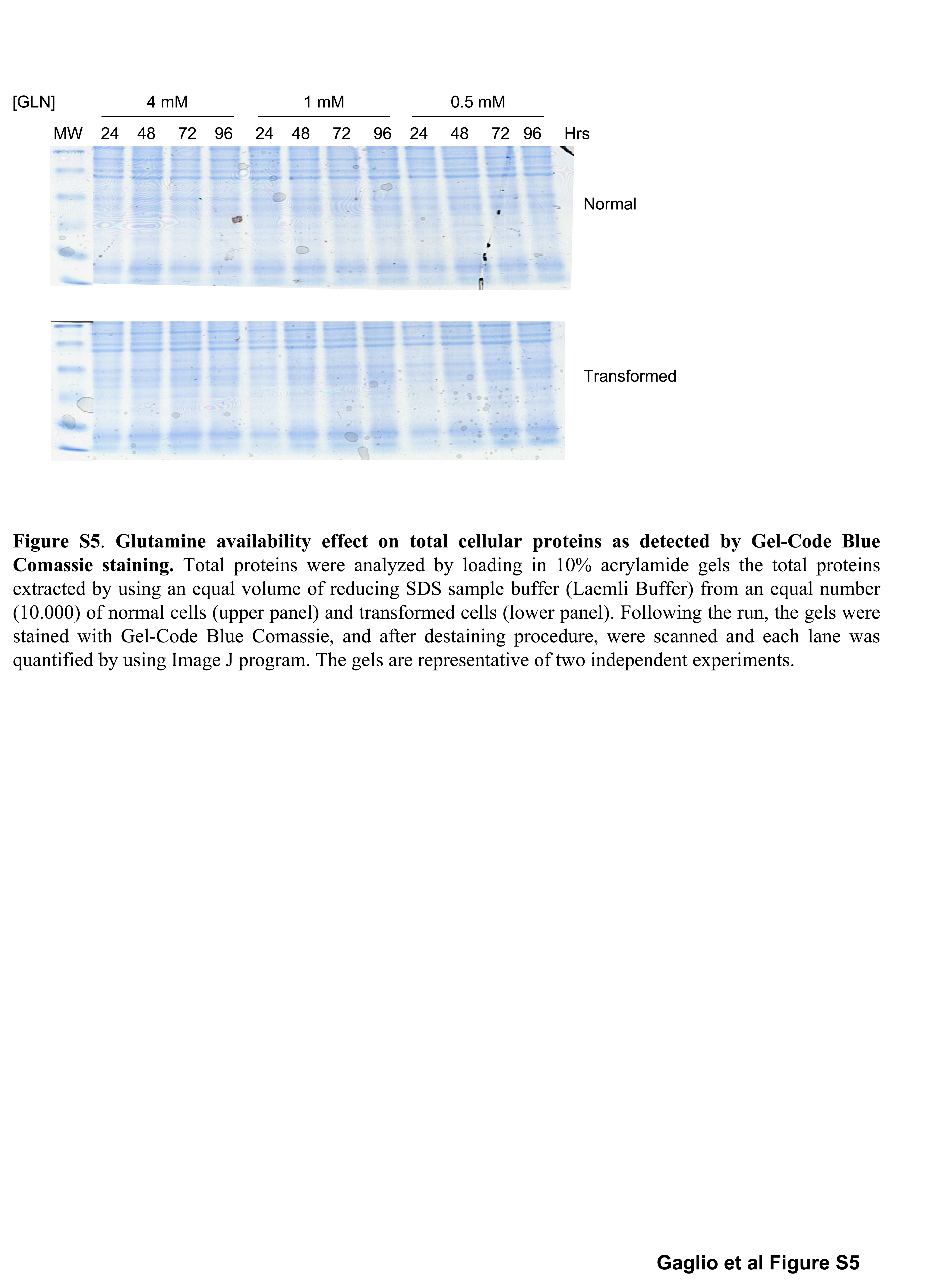

Supplement: Figure S5 — Glutamine availability effect on total cellular proteins as detected by Gel-Code Blue Comassie staining. (4.43 MB TIF) [file pone.0004715.s005.tif]

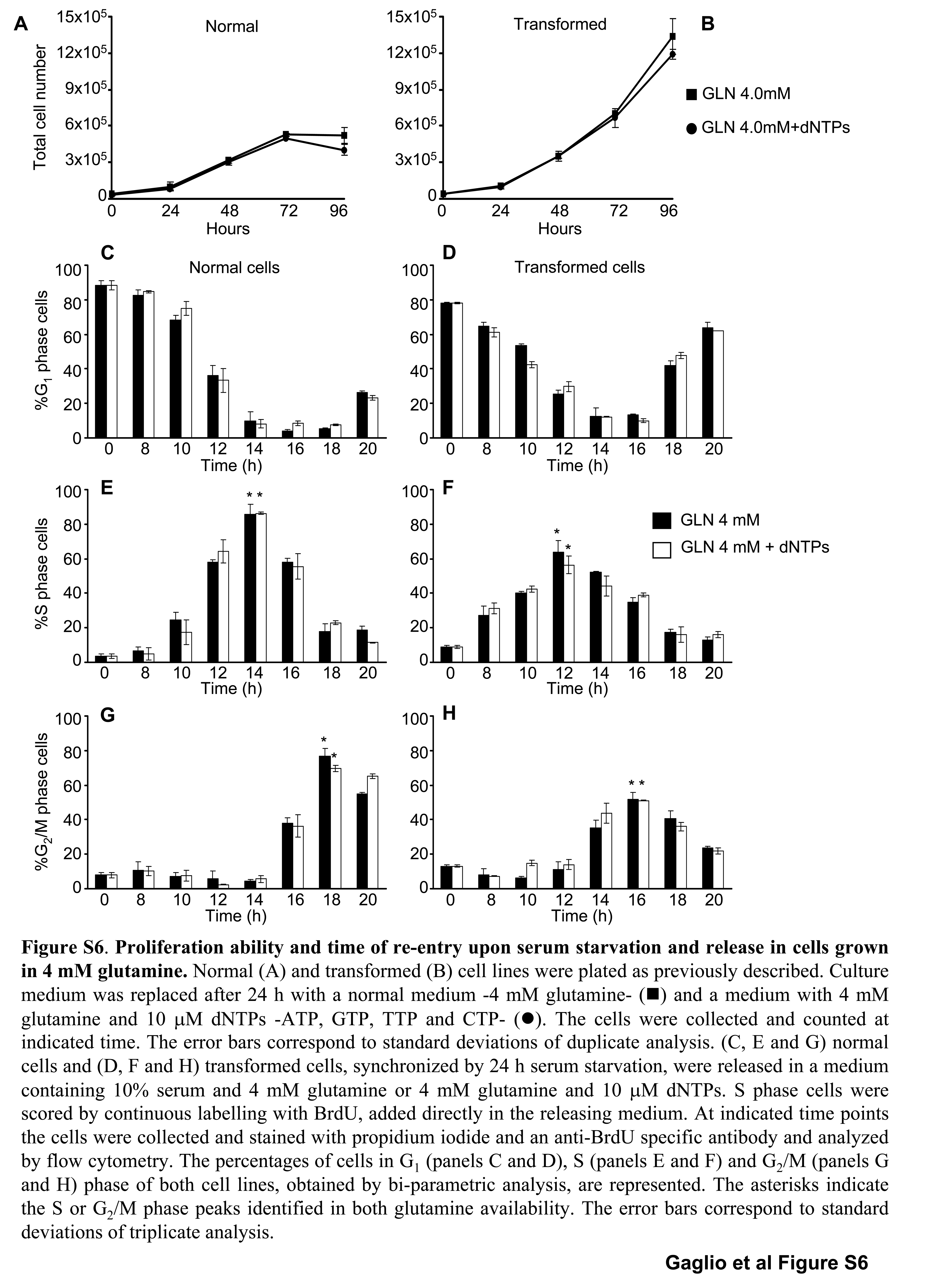

Supplement: Figure S6 — Proliferation ability and time of re-entry upon serum starvation and release in cells grown in 4 mM glutamine. (2.68 MB TIF) [file pone.0004715.s006.tif]

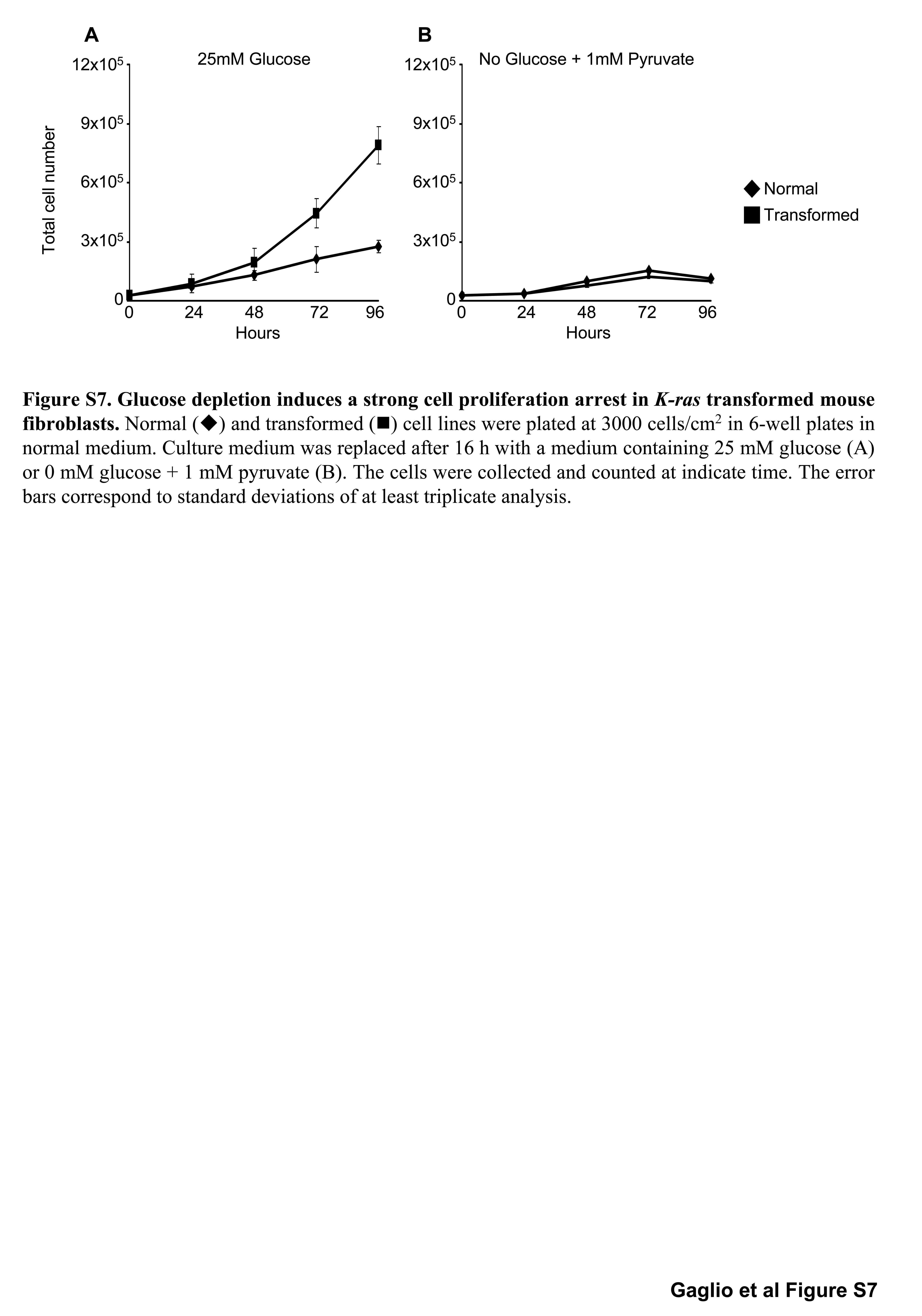

Supplement: Figure S7 — Glucose depletion induces a strong cell proliferation arrest in K-ras transformed mouse fibroblasts. (1.15 MB TIF) [file pone.0004715.s007.tif]
